# Clinical validation of ICNP^®^ Terminological Subset for people with chronic kidney disease undergoing hemodialysis

**DOI:** 10.1590/1980-220X-REEUSP-2025-0364en

**Published:** 2026-01-23

**Authors:** Richardson Augusto Rosendo da Silva, Juliana Otaciana dos Santos, Silvia Maria de Sá Basílio Lins, Harlon França de Menezes, Ana Beatriz Pereira da Silva, José Leonildo Fernandes de Queiroz, Rafaella Viesti Machado, Jéssica Fernanda Galon

**Affiliations:** 1 Universidade Federal do Rio Grande do Norte, Programa de Pós-graduação em Enfermagem, Natal, RN, Brazil.; 2 Universidade Federal Fluminense, Programa de Pós-graduação Mestrado Profissional em Enfermagem Assistencial, Niterói, RJ, Brazil.; 3 Universidade Federal do Rio Grande do Norte, Departamento de Enfermagem, Natal, RN, Brazil.

**Keywords:** Clinical Nursing Research, Validation Study, Standardized Nursing Terminology, Renal Dialysis, Renal Insufficiency, Chronic

## Abstract

**Objective:**

To clinically validate the nursing diagnoses/outcomes and interventions statements of the CIPE^®^ Subset for individuals with chronic kidney disease undergoing hemodialysis.

**Method:**

Clinical validation study, with individuals aged 18 years or older, with chronic kidney disease, undergoing dialysis for at least three months. The research took place in three stages: Application of the Nursing Process to clinical practice, encompassing five essential steps - Nursing assessment, diagnosis, planning, implementation, and progress notes; Structuring of case studies; and Identification of the prevalence of ND/NO and NI listed from the case studies. Data were analyzed using latent class analysis.

**Results:**

The findings revealed three distinct latent classes: low (Class 1), medium (Class 2), and high (Class 3) risk. The indicators showed high sensitivity (72.0% to 91.0%) and specificity (83.0% to 92.0%) values, confirming the clinical validity of the subset and the consistency of the model for discriminating different profiles of patients undergoing hemodialysis.

**Conclusion:**

The study clinically validated a subset of 23 nursing diagnoses/outcomes and 169 interventions, demonstrating their applicability in care.

## INTRODUCTION

The International Classification for Nursing Practice (ICNP^®^) plays a pivotal role in documenting nursing actions in clinical practice, ensuring standardization, organization, and greater accuracy in care records. This resource fosters clear and effective communication among healthcare professionals and facilitates the documentation of nursing care delivery^(1)^. The ICNP^®^ enables the development of Terminological Subsets, which comprise statements of nursing diagnoses/outcomes (ND/NO) and nursing interventions (NI) tailored to specific patient groups^(2)^.

Thus, the ICNP^®^ can be defined as a specialized communication system that represents nursing practice at both local and global levels, contributing to the effective application of the Nursing Process (NP). This standardized language constitutes an efficient strategy for the collection, storage, and analysis of nursing data, thereby strengthening and enhancing the visibility of the profession. It is noteworthy that, to improve the structuring of Terminological Subsets and increase their effectiveness, clinical validation is essential^(1)^.

For a more comprehensive structuring of this technology and to enhance its effectiveness, the application of a theoretical or conceptual model as a foundation for clinical practice is indispensable. Among the principal theoretical frameworks, the Basic Human Needs (BHN) model stands out as a cornerstone of nursing practice, enabling a holistic and multidimensional understanding of the human being. Originally proposed by Wanda Horta, these needs are classified into psychobiological, psychosocial, and psycho-spiritual dimensions, reflecting the physical, relational, and existential aspects of care. In the context of Chronic Kidney Disease (CKD) and hemodialysis therapy, BHN are frequently compromised, requiring a patient-centered care approach^(3)^.

According to data from the latest epidemiological bulletin on CKD in Brazil, it is estimated that approximately 10% of the Brazilian population has some degree of kidney impairment. In 2023, more than 187,000 primary healthcare visits related to CKD were recorded, predominantly among individuals aged 50 to 79^(1,2,3)^. The significant increase in hospitalizations for this condition, from 84,337 in 2010 to 140,648 in 2023, highlights the overload on healthcare services and the clinical complexity of patients undergoing hemodialysis^(4)^. This scenario reinforces the need for interventions based on models that consider the wholeness of the human being, such as the BHN theory, and tools that standardize professional language, such as the terminological subsets of an ICNP^^®^^.

Previous studies have already undertaken the development of terminology and the content validation of an ICNP^®^ terminological subset for individuals with CKD undergoing hemodialysis, guided by the BHN model. Its creation stemmed from the need to standardize nursing practice records and improve the quality of care documentation in hemodialysis services^(5,6)^.

In this regard, the Brazilian methodological framework encourages the clinical validation of terminological subsets to ensure their applicability and reliability in nursing practice. Clinical validation makes it possible to verify whether the statements developed adequately reflect the real conditions of individuals with CKD on hemodialysis, ensuring that the ND/NO and NI are effective in the clinical context. Moreover, this process allows for necessary adjustments, enhancing the precision of the terminologies used and promoting safer and more targeted care^(7)^.

The current state of the art regarding the use of ICNP^®^ in the care of individuals undergoing hemodialysis demonstrates that there remains a gap in the clinical validation of terminological subsets specific to this population. Nephrology nursing requires standardized instruments that enable effective communication among professionals, while also improving diagnostic accuracy and the implementation of targeted interventions. However, there is a lack of studies evaluating the applicability and reliability of ICNP^®^ statements in individuals with CKD on hemodialysis, which hinders their integration into clinical practice and the systematic documentation of care^(8,9,10)^.

In light of this gap, the present study seeks to address this need by clinically validating a terminological subset for individuals with chronic kidney disease undergoing hemodialysis, thereby providing scientific evidence to strengthen professional decision-making and contribute to patient safety and quality of care, justifying the relevance of this investigation.

The clinical significance of this study lies in offering important support for nursing care of hospitalized renal patients, by proposing a set of terms that more faithfully reflect their clinical needs. Furthermore, clinical validation strengthens evidence-based practice and may inform care protocols, nursing care plans, and educational strategies in nursing. In addition, it promotes the valorization of professional language and the visibility of nursing actions. Accordingly, this study aims to clinically validate the nursing diagnoses/outcomes and interventions of the ICNP^®^ subset for individuals with chronic kidney disease undergoing hemodialysis.

## METHOD

### Study Design and Local

This is a clinical validation study employing Latent Class Analysis (LCA)^(11)^, in which case studies were applied and conducted according to the phases established by the NP. The investigation was carried out in a renal outpatient clinic located in a municipality in the Northeast region of Brazil. Furthermore, the study was grounded in the Standards for Reporting Studies of Diagnostic Accuracy (STARD) initiative and diagnostic testing, with the objective of establishing the accuracy of the indicators^(1)^.

As the basis for clinical validation, the study utilized a terminological subset previously validated in terms of content in an earlier investigation, comprising 81 statements of ND, 242 statements of NO, and 533 statements of NI, structured according to the BHN theoretical framework^(5)^.

### Population, Selection Criteria, and Sample Definition

The sample size followed the recommendations of the latent class model, which requires a ratio of 5 to 30 participants per indicator assessed^(11)^. In the present study, a proportion of five patients was adopted for each of the 46 indicators evaluated, resulting in a final sample of 230 participants.

The sample consisted of individuals with CKD, aged 18 years or older, who had been undergoing dialysis treatment in the service for at least three months, according to the established inclusion criteria. Patients without cognitive capacity to participate in the study were excluded, as verified through information recorded in medical charts, such as medical and nursing notes describing dementia, neuropsychiatric disorders, or significant cognitive impairment; neuropsychological or psychiatric evaluation reports; and prescriptions of medications indicative of severe cognitive disorders.

Participant selection was conducted intentionally, based on chart screening and prior clinical assessment performed by the principal investigator. Patients were invited to perform simple cognitive tasks, such as stating their full name, date of birth, and recognizing the place and time of the interview. The objective was to ensure that all participants demonstrated full capacity for communication, comprehension, and consent, as required for the application of the Nursing Process and data collection.

### Data Collection

To achieve the established objective, the study was structured and conducted in three distinct phases: (1) application of the Nursing Process in clinical practice, encompassing five essential steps - assessment, diagnosis, planning, implementation, and nursing evaluation; (2) development of case studies; and (3) identification of the prevalence of ND/NO and NI derived from the case studies. The study was conducted between August 2024 and July 2025.

To guide data collection, a protocol was established that involved monitoring participants in two stages: the first consisted of a nursing consultation, followed by a second consultation focused on evaluation. No minimum interval was defined between consultations, as the protocol prioritized flexibility in follow-up, respecting individual availability and the specific demands of each case. This approach aimed to ensure greater adherence to the study and minimize potential sample losses without compromising the quality of the data collected. This stage was carried out between August 2024 and February 2025.

### First Stage

In the first phase, data collection, physical examination, and recordkeeping were performed by the principal investigator together with seven graduate students, using an instrument based on the BHN theory, thereby ensuring a structured and theoretically grounded approach.

The seven graduate students involved were nurses with prior experience in the use of the ICNP^®^ and the NP. They underwent specific and intensive training conducted by the principal investigator, which included a theoretical review of BHN, practical application of the data collection instrument, and clinical simulations focused on the identification of ND/NO and NI according to the ICNP^®^. This preparation aimed to ensure uniformity in the application of the instrument, conceptual fidelity to the adopted theoretical model, and quality in clinical records, guaranteeing that the data accurately reflected the care reality of patients undergoing hemodialysis.

The data collection instrument was structured into sections covering sociodemographic information, assessment, diagnosis, planning, implementation, and nursing evaluation. In addition, laboratory and imaging tests were analyzed to complement the clinical assessment.

Initially, a pilot test was conducted, previously approved by the Research Ethics Committee, with the objective of verifying the adequacy of the instrument. The participants in this pilot test were not included in the final sample, thereby ensuring the integrity of the data collected.

The ND/NO statements were established based on clinical indicators described in the operational definitions of the ICNP^®^ terminological subset. For organization and accessibility, these statements were systematized in an electronic form using Google Docs.

The planning and implementation phases were carried out in accordance with the NO established for each ND, as defined by the terminological subset. The proposed NI were directed toward meeting the identified needs, ensuring a personalized approach aligned with the objectives of care. The evaluation phase was conducted with a focus on the previously identified ND statements, allowing verification of the effectiveness of the interventions and the clinical progression of the participants.

### Second Stage

In the second phase, the researchers developed case studies, formulated the statements of ND/NO and NI, and identified their prevalence. These data were organized in a Microsoft Excel^®^ spreadsheet, structured into separate tabs, each corresponding to a patient. Subsequently, the statements were categorized according to psychobiological, psycho-spiritual, and psychosocial needs.

### Third Stage

In the final phase, latent class analysis was employed for the clinical validation of the terminological subset, based on patients with CKD undergoing hemodialysis, in order to identify clinically relevant patterns that supported the ICNP^®^ subset. During this stage, the ND/NO and NI identified in the patients, as described in the case studies, were considered clinically validated and incorporated into the terminological subset.

### Data Analysis and Treatment

The descriptive analysis included the calculation of absolute and relative frequencies of ND/NO, as well as the Shapiro-Wilk test. Due to the large number of statements, NI were grouped according to the categories of the theoretical model and discussed based on their frequency.

To ensure accuracy in data organization, all information was recorded in electronic spreadsheets. Within the context of this study, ND/NO and NI statements identified in patients, documented in case studies, and present in the terminological subset were considered clinically validated.

Clinical indicators were measured through interviews and physical examinations. Data were analyzed using the Statistical Package for the Social Sciences (SPSS) and R software (v.2.12.1). LCA was employed to calculate the sensitivity and specificity of the indicators. An indicator was considered statistically significant if at least one of its confidence intervals was greater than 0.5.

Variables were coded binarily (presence = 1; absence = 0). Modeling was conducted in R using the poLCA package. Models with two to five classes were tested, with the three-class model proving most appropriate, as indicated by the lowest Bayesian Information Criterion (BIC) value and entropy ≥ 0.80.

The latent profiles identified were interpreted based on the conditional frequency of ND/NO, allowing recognition of distinct clinical patterns, as described in the Results section. LCA identified three clinical groups with distinct profiles regarding the expression of nursing ND/NO: Class 1 (low risk): clinically compensated patients, with high treatment adherence and absence of significant physical or emotional symptoms; Class 2 (moderate risk): patients with low manifestation of physical signs but a strong prevalence of psychosocial diagnoses that could interfere with treatment adherence; Class 3 (high risk): multifactorially critical patients, with high frequency of physical symptoms, low treatment adherence, as well as spiritual distress and impaired religious bonds.

The classes were defined based on the presence of specific combinations of signs and symptoms/clinical indicators. Sensitivity indicated the model’s ability to correctly identify patients belonging to each class, whereas specificity assessed the extent to which the model avoided classifying individuals as belonging to a class when they did not fit its profile.

### Ethical Aspects

This study was conducted in accordance with the ethical principles of voluntariness, anonymity, and confidentiality, following the guidelines of Resolution No. 466/12 of the Brazilian National Health Council, which regulates research involving human subjects. The study was approved by the Research Ethics Committee of a federal institution of higher education, under approval number 7,753,717. To ensure voluntary and informed participation, all participants signed the Free and Informed Consent Form (FICF), thereby guaranteeing compliance with the applicable ethical and scientific standards.

## RESULTS

The sample consisted of 230 individuals undergoing hemodialysis due to chronic kidney disease, distributed across both sexes, with 41% female and 59% male. The majority were aged 60 years or older (86%), while only 14% were younger than 60 years. Regarding educational level, incomplete secondary education predominated (58%). With respect to marital status, 69% were married, and in terms of socioeconomic condition, 44% reported an income equal to or greater than two minimum wages. All participants presented at least one associated comorbidity, with arterial hypertension being the most prevalent (91%), followed by diabetes mellitus (81%).

In relation to the original subset, 23 ND/NO statements and 169 NI statements were implemented, with an average of 5 ND/NO statements per case study, ranging from a minimum of two to a maximum of 12.

Table 1 presents the diagnostic accuracy measures for each clinical indicator based on psychobiological human needs, including: Sensitivity (Se%) and its respective 95% confidence interval (95% CI) and Specificity (Sp%) with its 95% CI. These figures prove that the model of Latent Class Analysis generated robust estimates for clinical validation in these indicators.

**Table 1:** Frequency and diagnostic accuracy measures of clinical indicators for psychobiological human needs - Rio de Janeiro, RJ, Brazil, 2025.

Human need	Nursing diagnosis/outcome	Clinical indicator	Se (%) 95%CI	Sp (%) 95%CI
Oxygenation	Dyspnea Dyspnea, Absent	Respiratory rate ≥ 20 breaths per minute	0.94 (0.91–0.96)	0.88 (0.83–0.91)
		Peripheral O_2_ saturation < 92%	0.88 (0.84–0.91)	0.91 (0.87–0.94)
Hydration	Electrolyte imbalance/ Electrolyte balance, Adequate	Altered serum levels of sodium, potassium, and calcium.	0.86 (0.82–0.88)	0.89 (0.86–0.91)
		Presence of muscle cramps or weakness	0.85 (0.80–0.88)	0.90 (0.86–0.93)
Nutrition	Nutritional Intake, Impaired/ Nutritional Intake, Improved	Body mass index (BMI) outside the appropriate range.	0.90 (0.86–0.94)	0.95 (0.93–0.98)
		Abnormal laboratory tests (albumin, total proteins)	0.80 (0.75–0.84)	0.87 (0.82–0.90)
Voiding	Constipation/Constipation, Improved	No evacuation for more than 72 hours	0.94 (0.90–0.99)	0.91 (0.88–0.95)
		Hardened and dry stools	0.93 (0.90–0.97)	0.85 (0.82–0.88)
Sleep and rest	Sleep, Inadequate/Sleep, Adequate	Report of difficulty falling asleep.	0.90 (0.87–0.95)	0.94 (0.90–0.97)
		Use of sleep-inducing medication	0.82 (0.77–0.86)	0.86 (0.81–0.89)
Physical activity	Fatigue/Fatigue, Absent	Report of fatigue disproportionate to the effort.	0.87 (0.85–0.89)	0.94 (0.91–0.97)
		Need for frequent rest	0.83 (0.78–0.87)	0.88 (0.84–0.91)
Sexuality and reproduction	Sexual performance, Impaired/Sexual performance, Improved	Report of erectile dysfunction or anorgasmia	0.76 (0.71–0.80)	0.86 (0.81–0.89)
		Reduced sex drive	0.95 (0.90–0.99)	0.85 (0.81–0.88)
Physical and environmental security	Risk of Infection/Risk of Infection, Absent	Presence of an invasive device (catheter, venous access)	0.86 (0.81–0.89)	0.87 (0.82–0.90)
		Impaired skin integrity	0.90 (0.87–0.93)	0.89 (0.86–0.94)
Physical integrity	Skin integrity, Impaired/Skin integrity, Improved	Presence of pressure sores or ulcers	0.89 (0.85–0.93)	0.92 (0.90–0.94)
		Delayed wound healing	0.80 (0.75–0.84)	0.86 (0.81–0.89)
Vascular regulation	High Blood Pressure/Blood Pressure within Normal Limits	Blood pressure ≥ 140/90 mmHg at rest	0.91 (0.87–0.94)	0.86 (0.81–0.89)
		Edema in lower limbs	0.85 (0.80–0.87)	0.88 (0.85–0.92)
Thermal regulation	Fever/Fever, Absent	Body temperature > 37.8 °C	0.76 (0.71–0.80)	0.84 (0.79–0.88)
		Recurring chills	0.86 (0.83–0.90)	0.94 (0.90–0.98)
Neurological regulation	Memory, Impaired/Memory, Improved	Frequent forgetting of recent events	0.80 (0.75–0.84)	0.85 (0.80–0.88)
		Difficulty remembering simple instructions.	0.94 (0.91–0.96)	0.88 (0.83–0.91)
Hormonal regulation	Anemia, Chronic/Anemia Improved	Hemoglobin < 11 g/dL	0.84 (0.79–0.88)	0.88 (0.83–0.91)
		Skin and mucous membrane pallor	0.93 (0.89–0.96)	0.95 (0.93–0.98)
Sensory perception	Pain, Chronic/Chronic pain, Absent	Report of persistent pain > 3 months	0.84 (0.79–0.88)	0.87 (0.82–0.90)
		Continuous use of analgesics	0.91 (0.87–0.94)	0.84 (0.80–0.88)
Therapy and prevention	Non-adherence to hemodialysis treatment/adherence to Hemodialysis Treatment	Interdialytic weight gain exceeding the recommended level.	0.87 (0.84–0.90)	0.84 (0.80–0.87)
		Laboratory abnormalities due to non-adherence (urea, creatinine, potassium)	0.87 (0.83–0.91)	0.91 (0.88–0.94)

Legend: Se – sensitivity; Sp – specificity; 95% CI – 95% confidence interval.

The accuracy measures of the clinical indicators related to psychosocial and psycho-spiritual human needs are presented in Table 2.

**Table 2:** Frequency and diagnostic accuracy measures of clinical indicators by psychosocial and psycho-spiritual human needs - Rio de Janeiro, RJ, Brazil, 2025.

Human need	Nursing diagnosis/outcome	Clinical indicator	S (%) 95%CI	E (%) 95%CI
Gregariou (Social relations)	Impaired Family Process/Adequate Family Process	Isolation or withdrawal from family life	0.75 (0.70–0.79)	0.85 (0.80–0.88)
		Lack of emotional support reported by patient	0.97 (0.94–1.00)	0.91 (0.88–0.94)
Recreation and leisure	Impaired Leisure/Improved Leisure	Absence of recreational activities in routine	0.76 (0.71–0.80)	0,86 (0.81–0.89)
		Report of disinterest in leisure	0.86 (0.84–0.88)	0.90 (0.85–0.94)
Emotional security	Impaired Comfort/Improved Comfort	Report of general discomfort	0.80 (0.75–0.84)	0.84 (0.79–0.88)
		Facial expression of pain or discomfort	0.95 (0.91–0.97)	0.87 (0.84–0.92)
Self-esteem and self-respect	Negative Self-Esteem/Positive Self-Esteem	Report of self-depreciation	0.75 (0.70–0.79)	0.86 (0.81–0.89)
		Feeling of worthlessness	0.89 (0.87–0.92)	0.88 (0.86–0.91)
Freedom and participation	Treatment Denial/Treatment Acceptance	Verbal refusal of adherence to therapy	0.78 (0.73–0.82)	0.85 (0.80–0.88)
		Justifications for non-acceptance of clinical condition	0.97 (0.95–1.00)	0.87 (0.85–0.91)
Health education	Impaired Health Literacy/Adequate Health Literacy	Difficulty understanding medical prescriptions	0.79 (0.74–0.83)	0.86 (0.81–0.89)
		Inability to follow self-care instructions	0.93 (0.88–0.96)	0.83 (0.80–0.86)
Self-realization	Reduced Work Capacity/Improved Work Capacity	Report of work leave due to physical or emotional limitations	0.74 (0.69–0.78)	0.86 (0.81–0.89)
		Inability to perform usual occupational tasks	0.93 (0.89–0.96)	0,95 (0.93–0.98)
Religiosity and spirituality	Spiritual Distress/Absence of Distress	Anguish	0.73 (0.68–0.77)	0.86 (0.81–0.89)
		Feeling of abandonment by deity or faith community	0.86 (0.82–0.88)	0.86 (0.81–0.90)

Legend: Se – sensitivity; Sp – specificity; 95% CI – 95% confidence interval.

The analysis of the results demonstrates that the study achieved excellent performance in the clinical validation of interventions, as presented in Table 3. The three latent classes showed high sensitivity and specificity values, all above 80%, with narrow confidence intervals, reinforcing the statistical robustness and consistency of the findings.

**Table 3:** Examples of nursing interventions grouped by latent class, with diagnostic performance measures – Rio de Janeiro, RJ, Brazil, 2025.

Latent class	Predominant clinical profile	Priority interventions	S (%) 95%CI	E (%) 95%CI	F (%)
Class 1	Low risk	Monitor vital signs; Provide guidance on balanced diet; Promote treatment adherence; Strengthen family bonds; Reinforce skin care; Assess rest and sleep Quality.	82.5 (79.0–85.6)	87.2 (84.0–90.1)	36.8%
Class 2	Moderate risk	Encourage treatment adherence as a means of reducing future suffering; Assess pain and nausea; Stimulate self-care; Promote emotional expression; Encourage self-esteem, self-confidence, and disease acceptance; Provide physical and psychological comfort.	85.3 (82.1–88.0)	89.0 (86.2–91.5)	41.5%
Class 3	High risk	Manage respiratory and hemodynamic complications; Regulate diuresis, blood glucose, and blood pressure; Intensify neurological monitoring; Address spiritual distress; Reinforce the importance of regular hemodialysis to reduce mortality risk.	90.1 (87.5–92.4)	92.0 (89.8–94.0)	21.7%

Legend: Se – sensitivity; Sp – specificity; 95% CI – 95% confidence interval.

Class 1 comprised the largest proportion of the sample (36.8%), reflecting the predominant profile of patients under regular follow-up and with good treatment adherence. Class 2 represented 41.5% of the study population, confirming the relevance of educational and self-care interventions for this group. Class 3, although less prevalent (21.7%), presented the highest accuracy indices, indicating the precision of the model in identifying complex and more severe cases.

This overall performance suggests that the instrument used has high clinical applicability, contributing to greater effectiveness in the care of individuals undergoing hemodialysis. Due to page limitations, Table 3 presents examples of NI grouped by latent class, along with their respective diagnostic performance measures.

## DISCUSSION

The findings of this study demonstrate that the application of LCA with random effects was effective in the clinical validation of ICNP^®^ indicators, enabling the identification of diagnostic patterns with high sensitivity, specificity, and overall accuracy. This statistical approach reinforces the reliability of observable clinical indicators and their ability to discriminate distinct latent profiles, thereby contributing directly to the qualification of nursing diagnostic practice.

These results are consistent with a study that mapped evidence on the clinical validation of ICNP^®^ terminological subsets and identified that, although most research is still in its early stages, there is growing recognition of the importance of clinical validation as an essential step in consolidating standardized language in nursing practice^(1)^.

Furthermore, another study highlights that clinical validation contributes to improving the quality of care by ensuring that the terms used more accurately reflect the real needs of patients^(12)^. In the present study, the association between indicators and latent classes not only validated the terms but also enabled the grouping of specific interventions by clinical profile, representing a methodological advance compared to previous investigations, which focused primarily on content or semantic validation.

Within the domain of psychobiological needs, the ND/NO and NI statements demonstrated high clinical consistency and robustness, as evidenced by elevated sensitivity and specificity indices and narrow confidence intervals. These findings are in line with a methodological study that validated the ICNP^®^ subset for individuals undergoing hemodialysis, in which the majority of ND/NO and NI were concentrated in psychobiological needs, representing more than 70% of the validated statements^(5)^.

Similar results were observed in studies focused on the validation of specific interventions, such as fluid overload management, which showed high validation indices, reinforcing their clinical applicability in the care of patients receiving renal replacement therapy. Thus, the predominance of psychobiological needs in the findings of this investigation confirms the centrality of physiological aspects in the care process for hemodialysis patients, which are critical for maintaining clinical safety and treatment adherence^(13)^.

Within this domain, diagnoses reflecting respiratory, metabolic, and cardiovascular complications, such as Dyspnea and Electrolyte Imbalance, were validated, underscoring the importance of continuous monitoring and targeted interventions to ensure clinical stability. Other statements, such as Impaired Nutritional Intake, Constipation, and Inadequate Sleep, highlight the relevance of care directed toward nutritional, gastrointestinal, and rest dimensions, which are frequently compromised due to dietary restrictions, uremia, and associated symptoms. Additional validated diagnoses included Fatigue, Risk of Infection, Impaired Skin Integrity, Arterial Hypertension, Chronic Anemia, and Fever, all of which represent highly prevalent conditions in hemodialysis and demand strategies of prevention, health education, and treatment adherence^(14)^.

The diagnosis Impaired Memory may reflect cognitive alterations resulting from chronicity and impact adherence to clinical guidance, requiring greater educational support. Impaired Sexual Performance also emerged as relevant, often neglected in clinical practice, yet directly affecting self-esteem, interpersonal relationships, and quality of life. Finally, Non-Adherence to Hemodialysis Treatment was validated, a diagnosis that extends beyond mere attendance at sessions, encompassing acceptance, motivation, and coping with chronic illness. Recognition of these aspects broadens the perspective of care and reinforces the comprehensiveness of nursing assistance for individuals undergoing hemodialysis^(15,16,17)^.

In the domain of psychosocial needs, validated statements demonstrated moderate clinical consistency, although less prevalent compared to psychobiological ones. This finding aligns with other studies that identified a relevant, albeit minority, presence of ND/NO linked to psychosocial needs, representing approximately 20% of validated statements in terminological subsets^(5)^.

Highlighted diagnoses included Impaired Family Process, Impaired Leisure, and Impaired Comfort, which reflect the limitations imposed by the dialysis routine and associated symptoms, compromising family relationships, well-being, and quality of life. Additional diagnoses such as Negative Self-Esteem and Treatment Denial reveal emotional difficulties and challenges in accepting chronicity, while Impaired Health Literacy and Reduced Work Capacity illustrate the social and educational repercussions of the disease, with direct impacts on self-care autonomy and productivity. These findings reinforce the need for accessible educational interventions, psychological support, and strategies that promote daily life reorganization and social reintegration.

Patients undergoing hemodialysis frequently experience emotional impacts such as anxiety, fatigue, and impaired self-image, elements that directly influence their quality of life. Latent class clustering analysis applied to chronic diseases has shown that patient subgroups can be differentiated by the severity of psychosocial symptoms, underscoring the importance of interventions directed toward these needs as well^(18)^.

Thus, the inclusion of validated psychosocial statements expands the scope of care, moving beyond the physiological dimension and strengthening the comprehensiveness of nursing assistance^(19)^. Although fewer in number, the validation of statements related to psycho-spiritual needs represents an advance in recognizing the spiritual dimension in the care of individuals undergoing hemodialysis^(20)^.

Within the psycho-spiritual domain, the diagnosis Spiritual Distress highlighted the weight of the existential dimension in coping with chronic kidney disease. Dependence on continuous and invasive therapy may intensify feelings of emptiness, loss of meaning, and vulnerability, hindering adherence and coping. Validation of this statement points to the need for nursing practices that incorporate spirituality into care, valuing patients’ beliefs, values, and internal resources as strategies to strengthen resilience and promote comprehensive and humanized care.

In a clinical validation study with patients receiving conservative treatment, diagnoses such as Spiritual Distress and Impaired Religious Bond were confirmed as relevant, demonstrating that the spiritual dimension plays an important role in coping with chronic kidney disease^(16)^. Even though the proportion of such statements is small, their incorporation into the care plan reveals the sensitivity of ICNP^®^ in encompassing comprehensive and humanized care, addressing the existential experience of patients^(5,8,21)^.

The findings of this study reveal relevant convergences and contrasts when compared to the clinical validation of ICNP^®^ terminological subsets directed toward individuals with CKD under conservative treatment. The first concerns the predominance of ND/NO and NI related to treatment adherence, blood pressure control, and nutritional management. Although these elements are also present in the care of patients undergoing hemodialysis, the present study highlighted additional specificities, such as spiritual distress, emotional safety, and clinical burden resulting from dialysis-related complications. Moreover, the stratification of participants by latent classes enabled the identification of distinct care patterns, which had not been observed in studies involving patients under conservative treatment. These contrasts reinforce the importance of adapting terminological subsets to specific clinical realities, ensuring greater precision in the identification of BHN and in the personalization of nursing interventions^(9)^.

Therefore, the results obtained in this study not only clinically validate ICNP^®^ indicators but also expand their applicability by proposing a strategy of diagnostic stratification and intervention according to clinical profile. This proposal represents an innovative contribution to evidence-based practice and to the strengthening of professional nursing language.

The present analysis demonstrated the applicability of the BHN Theory, combined with LCA, as a robust method for identifying diagnostic patterns in patients undergoing hemodialysis. The adoption of a model with three latent classes enabled the clinical stratification of patients into distinct levels of complexity, providing relevant statistical support for personalized care planning.

The high sensitivity and specificity measures observed in several clinical indicators reinforce their discriminative capacity. Indicators associated with oxygenation, vascular regulation, nutrition, and elimination showed greater consistency in Class 3, evidencing that critical physiological aspects tend to be concentrated in patients with greater care needs. Conversely, indicators from psychosocial, emotional, and spiritual domains demonstrated adequate performance, particularly in Class 2, showing that such manifestations are not exclusive to severe cases and require transversal attention in nursing practice.

The grouping of interventions by latent class contributes directly to clinical practice by enabling the association between clinical profile and individualized care plans. The composition of interventions by class suggests that Class 1 patients benefit from educational and preventive actions; Class 2 patients require emotional support, symptom control, and reinforcement of self-care; and Class 3 patients demand complex and interdisciplinary plans for intensive management of clinical complications and psychosocial distress.

Thus, the results reinforce the potential of a multidimensional approach in the construction of DE/RE and IE, demonstrate the capacity of LCA to generate clinically applicable knowledge, and point to promising pathways for consolidating evidence-based practice within ICNP^®^ classification systems.

Regarding the limitations of the study, the selection of a specific population may have reduced the scope of the findings, making generalization more challenging. In addition, the restriction of the study to a single region and context introduced a unique cultural characteristic that must be carefully considered in the analysis of results, ensuring greater precision in their interpretation.

The implementation of the validated terminological subset for individuals undergoing hemodialysis requires a structured approach integrated into the routine of nursing professionals. First, it is essential to promote continuous training to enable nurses to correctly use the ND/NO and NI statements of the ICNP^®^. Workshops and clinical simulations may contribute to consolidating the clinical reasoning necessary for identifying the specific indicators of this subset’s diagnoses and for making assertive decisions.

Another essential aspect for successful implementation of the terminological subset is the creation of care protocols based on validated definitions. Incorporating these terms into hemodialysis practice guidelines can ensure greater uniformity in nursing practices and reduce variations in case management. Furthermore, the validation criteria may serve as a basis for internal audits, allowing monitoring of adherence to best practices and identification of opportunities for improvement in care delivery.

Finally, conducting multicenter studies constitutes a strategic step toward expanding the applicability of the validated subset in different healthcare institutions. Replicating the care model in other hemodialysis services will allow evaluation of its effectiveness in diverse contexts, reinforcing its utility in clinical practice. To ensure continuous updating, it is recommended that professionals involved participate in scientific collaboration networks, where they can share findings and contribute to the evolution of the ICNP^®^ terminological subset.

Effective implementation of this subset represents a significant advance in the standardization of specialized nursing language in hemodialysis, strengthening professional decision-making, enhancing patient safety, and ensuring higher-quality care for service users.

## CONCLUSION

The research highlighted the clinical validation of a subset composed of 23 ND/NO and 169 NI from ICNP^^®^^, directed toward the care of individuals with CKD undergoing hemodialysis. The findings demonstrate that the application of LCA constitutes a robust methodological approach for the clinical validation of ICNP^®^ terminological subsets, particularly in contexts characterized by high care complexity, such as hemodialysis.

The identification of three latent classes enabled the differentiation of distinct clinical profiles, with high levels of sensitivity, specificity, and overall accuracy, thereby reinforcing the validity of observable indicators for diagnostic purposes. In doing so, this study contributes to the consolidation of standardized nursing language, strengthens evidence-based practice, and provides support for the development of more precise, safe, and individualized care protocols.

The practical applicability of the subset is enhanced by its capacity to enable early risk identification, the planning of specific interventions, and continuous monitoring of nursing outcomes. Future research is recommended to explore its implementation in diverse care contexts, such as primary care, intensive care units, and renal transplant services, as well as to investigate its impact on care quality, clinical outcomes, and health service management.

## Data Availability

The entire dataset supporting the results of this study is available upon request to the corresponding author.

## References

[1] Ferreira FF, Lins SMSB, Menezes HF, Santos RSC, Santos JO, Silva RAR (2025). Clinical validation of terminological subsets of the international classification for nursing practice: a scoping review. Rev Bras Enferm.

[2] Menezes HF, Rosas AMMTF, Camacho ACLF, Souza FS, Rodrigues BMRD, Silva RAR (2018). Meaning of educational actions in nursing consultations for chronic renal clients and relatives. Rev Enferm UERJ.

[3] Souza TL, Trindade TRO, Mendonça AEO, Silva RAR (2016). Necessidades humanas básicas alteradas em pacientes pós-transplante renal: estudo transversal. Online Braz J Nurs.

[4] Brasil. (2024). Ministério da Saúde. Secretaria de Atenção Especializada à Saúde. Departamento de Atenção Especializada e Temática. Divulgado boletim epidemiológico sobre doença renal crônica no Brasil..

[5] Santos JO, Lins SMSB, Silva RAR, Menezes HF, Silva HCDA, Tavares JMAB (2024). Terminological subset of ICNP^®^ for people with chronic kidney disease on hemodialysis. Rev Esc Enferm USP.

[6] Santos JO, Lins SMSB, Nóbrega MML, Tavares JMAB, Menezes HF, Silva HCDA (2023). Specialized nursing terminology for chronic kidney patients on hemodialysis. Esc Anna Nery.

[7] Carvalho CMG, Cubas MR, Nóbrega MML (2017). Brazilian method for the development terminological subsets of ICNP^®^: limits and potentialities. Rev Bras Enferm.

[8] Menezes HF, Camacho ACLF, Lins SMSB, Campos TS, Lima FR, Jales AKFA (2020). Terms of specialized nursing language for chronic renal patients undergoing conservative treatment. Rev Bras Enferm.

[9] Menezes HF, Camacho ACLF, Monteiro PP, Santos IS, Pereira AB, Prado NCC (2024). Clinical validation of the terminological subset for people with chronic kidney disease undergoing conservative treatment. Rev Esc Enferm USP.

[10] Menezes HF, Camacho AC, Sant’Anna RM, Matos TL, Santos IS, Silva AB (2023). ICNP^
^®^
^ terminology subset for people with chronic kidney disease under conservative treatment. Acta Paul Enferm.

[11] Swanson SA, Lindenberg K, Bauer S, Crosby RD (2012). A Monte Carlo investigation of factors influencing latent class analysis: an application to eating disorder research. Int J Eat Disord.

[12] Olegário WKB, Fernandes LTB, Medeiros CMR (2015). Validation of ICNP^®^ Nursing Diagnoses for assistance to women during postpartum. Rev Eletr Enf.

[13] Lucena AF, Magro CZ, Proença MCC, Pires AUB, Moraes VM, Aliti GB (2017). Validação de intervenções e atividades de enfermagem para pacientes em terapia hemodialítica. Rev Gaúcha Enferm.

[14] Santos RSC, Bezerra MX, Castro RR, Menezes HF (2020). Content validation of a care plan for children with kidney diseases. Int J Dev Res.

[15] Menezes HF, Camacho AC, Sant’Anna RM, Matos TL, Santos IS, Silva AB (2023). ICNP^®^ terminology subset for people with chronic kidney disease under conservative treatment. Acta Paul Enferm.

[16] Menezes HF, Camacho ACLF, Sousa PAF, Primo CC, Ferreira LB, Silva RAR (2021). Validation of Nursing Diagnoses for people with chronic kidney conditions on conservative treatment. Rev Esc Enferm USP.

[17] Silva RA, Bezerra MX, Souza VL, Mendonça AE, Salvetti MG (2016). Nursing diagnoses, patient outcomes, and nursing interventions for patients undergoing peritoneal dialysis. Acta Paul Enferm.

[18] Lockwood MB, Lash JP, Pauls H, Chung SY, Samra M, Ryan C (2020). Physical symptom cluster subgroups in chronic kidney disease. Nurs Res.

[19] Menezes HF, Camacho ACLF, Nóbrega MML, Fuly PSC, Fernandes SF, Silva RAR (2020). Paths taken by Brazilian Nursing for the development of terminological subsets. Rev Latino-Am Enfermagem.

[20] Ferreira GSM, Fernandes PFCCB, Oliveira LC, Pinto JR, Ferreira IBM, Gurgel FF (2022). Religiosity, spirituality and quality of life in patients with chronic kidney disease who underwent hemodialysis in northeastern Brazil. RSD.

[21] Rocha CCT, Lima No AV, da Silva ABP, Farias VAS, D’Eça A, Silva RAR (2021). Nursing care for kidney transplant patients: a scoping review. Aquichan.

