# Erratum: Clinical validation of ICNP^®^ terminological subset for people with chronic kidney disease undergoing hemodialysis

**DOI:** 10.1590/1980-220X-REEUSP-2025-0364eren

**Published:** 2026-03-09

In the article “Clinical validation of ICNP^®^ terminological subset for people with chronic kidney disease undergoing hemodialysis”, with DOI code number: https://doi.org/10.1590/1980-220X-REEUSP-2025-0364en, published in the journal Revista da Escola de Enfermagem da USP, 60:e20250364, 2026, in the page 1:

**Where it was written:**

“Juliana Otaciana da Silva”

**Should read:**

“Juliana Otaciana dos Santos”

Prof. Dr. Maria Amélia de Campos Oliveira

Editor-in-chief

