# Supplementary material for: Clinical validation of ICNP® Terminological Subset for people with chronic kidney disease undergoing hemodialysis
Source: Rev Esc Enferm USP. 2026 Jan 23;60:e20250364. doi: 10.1590/1980-220X-REEUSP-2025-0364en (PMC12854694; doi:10.1590/1980-220X-REEUSP-2025-0364en)
Supplement: Translation

# Translation: Validação clínica do Subconjunto Terminológico da CIPE^®^ para pessoas com doença renal crônica em hemodiálise

**DOI:** 10.1590/1980-220X-REEUSP-2025-0364pt

**Authors:** Richardson Augusto Rosendo da Silva, Juliana Otaciana dos Santos, Silvia Maria de Sá Basílio Lins, Harlon França de Menezes, Ana Beatriz Pereira da Silva, José Leonildo Fernandes de Queiroz, Rafaella Viesti Machado, Jéssica Fernanda Galon

**Affiliations:** 1 Universidade Federal do Rio Grande do Norte, Programa de Pós-graduação em Enfermagem, Natal, RN, Brasil.; 2 Universidade Federal Fluminense, Programa de Pós-graduação Mestrado Profissional em Enfermagem Assistencial, Niterói, RJ, Brasil.; 3 Universidade Federal do Rio Grande do Norte, Departamento de Enfermagem, Natal, RN, Brasil.

**Keywords:** Pesquisa em Enfermagem Clínica, Estudo de Validação, Terminologia Padronizada em Enfermagem, Diálise Renal, Insuficiência Renal Crônica

## Abstract

**Objetivo:**

Validar clinicamente os enunciados de diagnósticos/resultados e intervenções de enfermagem do Subconjunto CIPE^®^ para pessoas com doença renal crônica em hemodiálise.

**Método:**

Estudo de validação clínica, com indivíduos de idade igual ou superior a 18 anos, doentes renais crônicos, em tratamento dialítico há pelo menos três meses. A pesquisa ocorreu em três etapas: Aplicação do Processo de Enfermagem à prática clínica, abrangendo cinco etapas essenciais – avaliação, diagnóstico, planejamento, implementação e evolução de Enfermagem; Estruturação dos estudos de casos; e Identificação da prevalência dos DE/RE e IE elencados a partir dos estudos de casos. Os dados foram analisados utilizando-se a análise de classe latente.

**Resultados:**

Os achados evidenciaram três classes latentes distintas: baixo (Classe 1), médio (Classe 2) e alto risco (Classe 3). Os indicadores apresentaram elevados valores de sensibilidade (72,0% a 91,0%) e especificidade (83,0% a 92,0%), confirmando a validade clínica do subconjunto e a consistência do modelo para discriminar diferentes perfis de pacientes em hemodiálise.

**Conclusão:**

O estudo validou clinicamente um subconjunto de 23 diagnósticos/resultados e 169 intervenções de enfermagem, demonstrando aplicabilidade na prática assistencial.

## INTRODUÇÃO

A Classificação Internacional para a Prática de Enfermagem (CIPE^®^) desempenha papel essencial na documentação das ações do enfermeiro na prática clínica, garantindo padronização, organização e maior precisão nos registros assistenciais. Esse recurso favorece uma comunicação clara e eficaz entre os profissionais de saúde, além de facilitar a documentação da assistência prestada^(1)^. A CIPE^®^ possibilita o desenvolvimento de Subconjuntos Terminológicos, que reúnem enunciados de diagnósticos/resultados (DE/RE) e intervenções de enfermagem (IE), direcionados a grupos específicos de pacientes^(2)^.

Assim, pode-se definir a CIPE^®^ como um sistema de comunicação especializado e representativo da prática de enfermagem, com alcance local e global, contribuindo para a aplicação eficaz do Processo de Enfermagem (PE). Essa linguagem constitui uma estratégia eficiente para a coleta, o armazenamento e a análise de dados em enfermagem, favorecendo o fortalecimento e a valorização da profissão. Destaca-se que, para aprimorar a estruturação dos Subconjuntos Terminológicos e aumentar sua eficácia, torna-se essencial validá-los no contexto clínico^(1)^.

Para uma melhor estruturação dessa tecnologia e para torná-la mais eficaz, é fundamental a aplicação de um modelo teórico ou conceitual que sirva de base à prática clínica. Entre os principais modelos teóricos, destaca-se o das Necessidades Humanas Básicas (NHB), que constitui o alicerce da prática de enfermagem, permitindo compreender o ser humano de forma integral e multidimensional. Propostas originalmente por Wanda Horta, essas necessidades são classificadas em psicobiológicas, psicossociais e psicoespirituais, refletindo as dimensões física, relacional e existencial do cuidado. No contexto da Doença Renal Crônica (DRC) e da terapia hemodialítica, as NHB são frequentemente comprometidas, exigindo uma abordagem assistencial centrada no indivíduo^(3)^.

Segundo dados do último boletim epidemiológico sobre DRC no Brasil, estima-se que cerca de 10% da população apresenta algum grau de comprometimento renal. Em 2023, foram registrados mais de 187 mil atendimentos na Atenção Primária à Saúde relacionados à DRC, com predominância entre indivíduos de 50 a 79 anos^(1-3)^. O aumento expressivo nas internações por essa condição, de 84.337 em 2010 para 140.648 em 2023, evidencia a sobrecarga nos serviços de saúde e a complexidade clínica dos pacientes em hemodiálise^(4)^. Esse cenário reforça a necessidade de intervenções fundamentadas em modelos que considerem a integralidade do ser humano, como o das NHB, e de ferramentas que padronizem a linguagem profissional, como os Subconjuntos Terminológicos da CIPE^®^.

Estudos prévios já realizaram a construção da terminologia e a validação do conteúdo de um subconjunto terminológico da CIPE^®^ para pessoas com doença renal crônica em hemodiálise, à luz do modelo das NHB. Sua elaboração surgiu da necessidade de padronizar os registros da prática de enfermagem e aprimorar a qualidade da documentação assistencial em serviços de hemodiálise^(5,6)^.

Nesse sentido, o método brasileiro encoraja a validação clínica dos subconjuntos, a fim de garantir sua aplicabilidade e confiabilidade na prática assistencial. A validação clínica possibilita verificar se os enunciados desenvolvidos refletem adequadamente as condições reais das pessoas com DRC em hemodiálise, assegurando que os DE/RE e IE sejam eficazes no contexto clínico. Além disso, esse processo permite ajustes necessários, aprimorando a precisão das terminologias utilizadas e favorecendo um cuidado mais direcionado e seguro^(7)^.

O estado da arte sobre a utilização da CIPE^®^ no cuidado às pessoas em hemodiálise demonstra que ainda há uma lacuna quanto à validação clínica de subconjuntos terminológicos específicos para essa população. A enfermagem em nefrologia demanda instrumentos padronizados que possibilitem uma comunicação eficaz entre profissionais, além de melhorar a precisão diagnóstica e a implementação de intervenções direcionadas. No entanto, observa-se ausência de estudos que avaliaram a aplicabilidade e a confiabilidade dos enunciados da CIPE^®^ em pessoas com DRC em hemodiálise, o que dificulta sua integração na prática clínica e na documentação sistematizada do cuidado^(8-10)^.

Diante dessa lacuna, esta pesquisa busca preencher tal necessidade ao validar clinicamente um subconjunto terminológico para pessoas com doença renal crônica em hemodiálise, oferecendo embasamento científico que fortaleça a tomada de decisão dos profissionais e contribua para a segurança e qualidade da assistência prestada, justificando a realização do presente estudo.

A relevância clínica do estudo reside em oferecer subsídios importantes para a assistência de enfermagem a pacientes renais hospitalizados, ao propor um conjunto de termos que reflita com maior fidelidade as necessidades clínicas desses pacientes. Além disso, a validação clínica fortalece a prática baseada em evidências e pode subsidiar protocolos assistenciais, planos de cuidado e o ensino em enfermagem. Ademais, promove a valorização da linguagem profissional e a visibilidade das ações de enfermagem. Logo, este estudo tem como objetivo validar clinicamente os enunciados de diagnósticos/resultados e intervenções de enfermagem do Subconjunto CIPE^®^ para pessoas com doença renal crônica em hemodiálise.

## MÉTODO

### Desenho e Local do Estudo

Trata-se de um estudo de validação clínica, utilizando a Análise de Classes Latentes (ACL)^(11)^, no qual foram aplicados estudos de caso conduzidos de acordo com as fases estabelecidas pelo Processo de Enfermagem (PE). A investigação foi realizada em um ambulatório de doenças renais situado em um município da região Nordeste do Brasil. Além disso, o estudo fundamentou-se na iniciativa *Standards for Reporting Studies of Diagnostic Accuracy* (STARD) e em testes diagnósticos, com o objetivo de estabelecer a acurácia dos indicadores^(1)^.

Como base para a validação clínica, utilizou-se o subconjunto terminológico previamente validado em relação ao conteúdo em estudo anterior, composto por 81 enunciados de DE, 242 enunciados de RE e 533 enunciados de IE, à luz da Teoria das NHB^(5)^.

### População, Critérios de Seleção e Definição da Amostra

O tamanho da amostra seguiu as recomendações do modelo de classe latente, que prevê uma relação de 5 a 30 participantes por indicador avaliado^(11)^. No presente estudo, adotou-se a proporção de cinco pacientes para cada um dos 46 indicadores avaliados, obtendo-se uma amostra final de 230 participantes.

A amostra foi composta por indivíduos com doença renal crônica, com idade igual ou superior a 18 anos e em tratamento dialítico no serviço há pelo menos três meses, conforme critérios de inclusão estabelecidos. Foram excluídos os pacientes sem capacidade cognitiva para participar da pesquisa, verificada a partir das informações registradas nos prontuários, tais como: evoluções médicas e de enfermagem que descreviam quadros de demência, transtornos neuropsiquiátricos ou comprometimento cognitivo significativo; laudos de avaliação neuropsicológica ou psiquiátrica; e prescrição de medicamentos indicativos de distúrbios cognitivos severos.

A seleção dos participantes foi realizada de forma intencional, com base na triagem dos prontuários e na avaliação clínica prévia conduzida pelo pesquisador responsável. Os pacientes eram convidados a realizar pequenas tarefas cognitivas, como informar nome completo, data de nascimento e reconhecer o local e o momento da entrevista. O objetivo foi garantir que todos os participantes apresentassem plena capacidade de comunicação, compreensão e consentimento, conforme exigido para a aplicação do Processo de Enfermagem e coleta de dados.

### Coleta de Dados

Para alcançar o objetivo estabelecido, o estudo foi estruturado e conduzido em três fases distintas: 1º) Aplicação do Processo de Enfermagem à prática clínica, abrangendo cinco etapas essenciais - avaliação, diagnóstico, planejamento, implementação e evolução de enfermagem; 2º) Estruturação dos estudos de caso; 3º) Identificação da prevalência dos DE/RE e IE elencados a partir dos estudos de caso. O estudo foi conduzido entre agosto de 2024 e julho de 2025.

Para orientar a coleta de dados, estabeleceu-se um protocolo que previa o acompanhamento dos participantes em duas etapas: a primeira consistia em uma consulta de enfermagem, seguida por uma segunda consulta destinada à avaliação. Não foi definido intervalo mínimo entre as consultas, uma vez que o protocolo priorizou a flexibilidade no acompanhamento, respeitando a disponibilidade individual e as demandas específicas de cada caso. Essa abordagem visou garantir maior adesão ao estudo e minimizar possíveis perdas amostrais, sem comprometer a qualidade dos dados coletados. Essa etapa ocorreu entre agosto de 2024 e fevereiro de 2025.

### Primeira Etapa

Na primeira fase, a coleta de dados, o exame físico e os registros foram realizados pelo pesquisador principal e por sete estudantes de pós-graduação, com o suporte de um instrumento baseado na Teoria das NHB, garantindo uma abordagem estruturada e fundamentada.

Os sete estudantes de pós-graduação envolvidos eram enfermeiros com experiência no uso da CIPE^®^ e do PE, e passaram por treinamento específico e intensivo conduzido pelo pesquisador principal. Esse treinamento incluiu revisão teórica sobre as NHB, aplicação prática do instrumento de coleta e simulações clínicas com foco na identificação de DE/RE e IE segundo a CIPE^®^. O preparo visou assegurar uniformidade na aplicação do instrumento, fidelidade conceitual ao modelo teórico adotado e qualidade nos registros clínicos, garantindo que os dados refletissem com precisão a realidade assistencial dos pacientes em hemodiálise.

O instrumento de coleta foi estruturado em seções que contemplavam dados sociodemográficos, avaliação, diagnóstico, planejamento, implementação e evolução de enfermagem. Além disso, foram analisados exames laboratoriais e de imagem para complementar a avaliação clínica.

Inicialmente, realizou-se um teste-piloto, previamente aprovado pelo Comitê de Ética em Pesquisa, com o objetivo de verificar a adequação do instrumento. Os participantes desse teste não foram incluídos na amostra final, garantindo a integridade dos dados coletados.

Os enunciados de DE/RE foram estabelecidos com base em indicadores clínicos descritos nas definições operacionais do subconjunto terminológico da CIPE^®^. Para organização e acessibilidade, esses enunciados foram sistematizados em formulário eletrônico no Google Docs.

As etapas de planejamento e implementação foram realizadas em conformidade com os RE estabelecidos para cada DE, conforme definido pelo subconjunto terminológico. As IE propostas foram orientadas para atender às necessidades identificadas, assegurando abordagem personalizada e alinhada aos objetivos do cuidado. Já a etapa de avaliação foi conduzida com foco nos enunciados de DE previamente identificados, permitindo verificar a efetividade das intervenções e a evolução clínica dos participantes.

### Segunda Etapa

Na segunda fase, os pesquisadores desenvolveram os estudos de caso, formularam os enunciados de DE/RE e IE e identificaram sua prevalência. Essas informações foram organizadas em planilha do Microsoft Excel^®^, estruturada em abas separadas, cada uma correspondente a um paciente. Posteriormente, os enunciados foram categorizados conforme as necessidades psicobiológicas, psicoespirituais e psicossociais.

### Terceira Etapa

Na fase final, empregou-se a análise de classes latentes para a validação clínica do subconjunto terminológico, com base nos pacientes com DRC em hemodiálise, de modo a verificar padrões clínicos relevantes que sustentassem o subconjunto da CIPE^®^. Durante essa etapa, foram considerados clinicamente validados os DE/RE e IE identificados nos pacientes, conforme descritos nos estudos de caso, os quais foram incorporados ao subconjunto terminológico.

### Análise e Tratamento dos Dados

A análise descritiva incluiu o cálculo de frequências absolutas e percentuais dos enunciados de DE/RE, além do teste de Shapiro-Wilk. Devido ao grande número de enunciados, as IE foram agrupadas conforme a categoria do modelo teórico e discutidas com base na frequência.

Para garantir precisão na organização dos dados, todas as informações foram registradas em planilha eletrônica. No contexto desta pesquisa, foram considerados clinicamente validados os enunciados de DE/RE e IE identificados nos pacientes, documentados nos estudos de caso e presentes no subconjunto terminológico.

Os indicadores clínicos foram mensurados por meio de entrevista e exame físico. Os dados foram analisados com o auxílio dos softwares *Statistical Package for the Social Sciences* (SPSS) e R (v. 2.12.1). O método de análise de classes latentes foi utilizado para calcular a sensibilidade e especificidade dos indicadores. O indicador foi considerado estatisticamente significativo se pelo menos um de seus intervalos de confiança fosse >0,5.

As variáveis foram codificadas binariamente (presença = 1; ausência = 0). A modelagem foi conduzida com o software R, utilizando o pacote *poLCA*. Foram testados modelos com 2 a 5 classes, sendo o modelo com três classes o mais adequado, conforme menor valor do BIC (*Bayesian Information Criterion*) e entropia ≥ 0,80.

Os perfis latentes identificados foram interpretados com base na frequência condicional dos DE/RE, permitindo reconhecer padrões clínicos distintos, descritos na seção de Resultados. A ACL identificou três grupos clínicos com perfis distintos quanto à expressão dos DE/RE de enfermagem: Classe 1 (baixo risco): pacientes clinicamente compensados, com alta adesão ao tratamento e ausência de sintomas físicos ou emocionais significativos; Classe 2 (médio risco): baixa manifestação de sinais físicos, mas forte prevalência de DE de natureza psicossocial que poderiam interferir na adesão ao tratamento; Classe 3 (alto risco): pacientes críticos multifatoriais, com alta frequência de sintomas físicos, baixa adesão ao tratamento, além de sofrimento espiritual e prejuízos nos laços religiosos.

As classes foram definidas a partir da presença de combinações específicas de sinais e sintomas/indicadores clínicos. A sensibilidade indicou a capacidade do modelo de identificar corretamente os pacientes pertencentes a cada classe, enquanto a especificidade avaliou o quanto o modelo evitou classificar como pertencentes à classe indivíduos que não se enquadravam nela.

### Aspectos Éticos

O presente estudo foi conduzido em conformidade com os princípios éticos de voluntariedade, anonimato e confidencialidade, atendendo às diretrizes da Resolução nº 466/12 do Conselho Nacional de Saúde, que regulamenta pesquisas envolvendo seres humanos. A pesquisa foi aprovada pelo Comitê de Ética em Pesquisa de uma instituição federal de ensino superior, conforme parecer nº 7.753.717. Para assegurar a participação voluntária e esclarecida, todos os participantes assinaram o Termo de Consentimento Livre e Esclarecido (TCLE), garantindo o cumprimento das normativas éticas e científicas aplicáveis.

## RESULTADOS

A amostra foi composta por 230 indivíduos em tratamento hemodialítico devido à doença renal crônica, distribuídos entre ambos os sexos, sendo 41% do sexo feminino e 59% do masculino. A maioria apresentava idade igual ou superior a 60 anos (86%), enquanto apenas 14% tinha menos de 60 anos. Em relação à escolaridade, predominou o ensino médio incompleto (58%). Quanto ao estado civil, 69% eram casados e, no que se refere à condição socioeconômica, 44% relataram renda igual ou superior a dois salários mínimos. Observou-se que todos possuíam alguma comorbidade associada, sendo a hipertensão arterial a mais prevalente (91%), seguida pelo diabetes mellitus (81%).

No que diz respeito ao subconjunto original, foram implementados 23 enunciados de DE/RE e 169 IE, com média de cinco enunciados de DE/RE por estudo de caso, variando entre um mínimo de dois e um máximo de 12.

A Tabela 1 apresenta as medidas de acurácia diagnóstica para cada indicador clínico, organizadas segundo as necessidades humanas psicobiológicas. Incluem-se: Sensibilidade (Se%) e seus respectivos intervalos de confiança de 95% (IC95%), bem como Especificidade (Es%) com seus IC95%. Esses valores demonstram que o modelo de Análise de Classes Latentes gerou estimativas robustas para a validação clínica desses indicadores.

**Tabela 1:** Frequência e medidas de acurácia diagnóstica dos indicadores clínicos por necessidades humanas psicobiológicas – Rio de Janeiro, RJ, Brasil, 2025.

Necessidade humana	Diagnóstico/resultado de enfermagem	Indicador clínico	S (%) IC95%	E (%) IC95%
Oxigenação	Dispneia/Dispneia, Ausente	Frequência respiratória ≥ 20 irpm	0,94 (0,91–0,96)	0,88 (0,83–0,91)
		Saturação periférica de O_2_ < 92%	0,88 (0,84–0,91)	0,91 (0,87–0,94)
Hidratação	Desequilíbrio de eletrólitos/Equilíbrio de eletrólitos, Adequado	Níveis séricos alterados de sódio, potássio e cálcio	0,86 (0,82–0,88)	0,89 (0,86–0.91)
		Presença de cãibras ou fraqueza muscular	0,85 (0,80–0,88)	0,90 (0,86–0,93)
Nutrição	Ingestão Nutricional, Prejudicada/Ingestão Nutricional, Melhorada	Índice de massa corporal (IMC) fora da faixa adequada	0,90 (0,86–0,94)	0,95 (0,93–0,98)
		Exames laboratoriais alterados (albumina, proteínas totais)	0,80 (0,75–0,84)	0,87 (0,82–0,90)
Eliminação	Constipação/Constipação, Melhorada	Evacuação ausente por mais de 72 horas	0,94 (0,90–0,99)	0,91 (0,88–0,95)
		Fezes endurecidas e ressecadas	0,93 (0,90–0,97)	0,85 (0,82–0,88)
Sono e repouso	Sono, Inadequado/Sono, Adequado	Relato de dificuldade para iniciar o sono	0.90 (0.87–0.95)	0.94 (0.90–0.97)
		Uso de medicação indutora de sono	0,82 (0,77–0,86)	0,86 (0,81–0,89)
Atividade física	Fadiga/Fadiga, Ausente	Relato de cansaço desproporcional ao esforço	0,87 (0,85–0,89)	0,94 (0,91–0,97)
		Necessidade de repouso frequente	0,83 (0,78–0,87)	0,88 (0,84–0,91)
Sexualidade e reprodução	Desempenho sexual, Prejudicado/Desempenho sexual, Melhorado	Relato de disfunção erétil ou anorgasmia	0,76 (0,71–0,80)	0,86 (0,81–0,89)
		Redução do desejo sexual	0,95 (0,90–0,99)	0,85 (0,81–0,88)
Segurança física e ambiental	Risco de Infecção/Risco de Infecção, Ausente	Presença de dispositivo invasivo (cateter, acesso venoso)	0,86 (0,81–0,89)	0,87 (0,82–0,90)
		Integridade cutânea prejudicada	0,90 (0,87–0,93)	0,89 (0,86–0,94)
Integridade física	Integridade da pele, Prejudicada/Integridade da pele, Melhorada	Presença de lesões ou úlceras de pressão	0,89 (0,85–0,93)	0,92 (0,90–0,94)
		Retardo na cicatrização de feridas	0,80 (0,75–0,84)	0,86 (0,81–0,89)
Regulação vascular	Hipertensão Arterial/Pressão Arterial, nos Limites Normais	Pressão arterial ≥ 140/90 mmHg em repouso	0,91 (0,87–0,94)	0,86 (0,81–0,89)
		Edema em membros inferiores	0,85 (0,80–0.87)	0,88 (0,85–0,92)
Regulação térmica	Febre/Febre, Ausente	Temperatura corporal > 37,8 °C	0,76 (0,71–0,80)	0,84 (0,79–0,88)
		Calafrios recorrentes	0,86 (0,83–0,90)	0,94 (0,90–0,98)
Regulação neurológica	Memória, Prejudicada/Memória, Melhorada	Esquecimento frequente de fatos recentes	0,80 (0,75–0,84)	0,85 (0,80–0,88)
		Dificuldade em recordar instruções simples	0,94 (0.91–0.96)	0,88 (0,83–0,91)
Regulação hormonal	Anemia, Crônica/Anemia, Melhorada	Hemoglobina < 11 g/dL	0,84 (0,79–0,88)	0,88 (0,83–0,91)
		Palidez cutâneo-mucosa	0,93 (0.89–0,96)	0,95 (0,93–0,98)
Sensopercepção	Dor, Crônica/Dor Crônica, Ausente	Relato de dor persistente > 3 meses	0,84 (0,79–0,88)	0,87 (0,82–0,90)
		Uso contínuo de analgésicos	0.91 (0,87–0,94)	0,84 (0,80–0,88)
Terapêutica e prevenção	Não Adesão ao Tratamento Hemodialítico/Adesão ao Tratamento Hemodialítico	Ganho de peso interdialítico acima do recomendado	0,87 (0,84–0,90)	0,84 (0,80–0,87)
		Alterações laboratoriais por não adesão (ureia, creatinina, potássio)	0,87 (0,83–0,91)	0,91 (0,88–0,94)

Legenda: Se – sensibilidade; Es – especificidade; IC 95% – intervalo de confiança de 95%.

As medidas de acurácia dos indicadores clínicos, referentes às necessidades humanas psicossociais e psicoespirituais, estão apresentadas na Tabela 2.

**Tabela 2:** Frequência e medidas de acurácia diagnóstica dos indicadores clínicos por necessidade humana psicossociais e psicoespirituais - Rio de Janeiro, RJ, Brasil, 2025.

Necessidade humana	Diagnóstico/resultado de enfermagem	Indicador clínico	S (%) IC95%	E (%) IC95%
Gregária (relações sociais)	Processo Familiar, Prejudicado/Processo Familiar, Adequado	Isolamento ou afastamento do convívio familiar	0,75 (0.70–0,79)	0,85 (0,80–0,88)
		Falta de apoio emocional relatada pelo paciente	0,97 (0,94–1,00)	0,91 (0,88–0,94)
Recreação e lazer	Lazer prejudicado/Lazer Melhorado	Ausência de atividades recreativas na rotina	0,76 (0,71–0,80)	0,86 (0,81–0,89)
		Relato de desinteresse por lazer	0,86 (0,84–0,88)	0,90 (0,85–0,94)
Segurança emocional	Conforto prejudicado/Conforto Melhorado	Relato de mal-estar geral	0,80 (0,75–0,84)	0,84 (0,79–0,88)
		Expressão facial de dor ou desconforto	0,95 (0,91–0,97)	0,87 (0,84–0,92)
Autoestima e autorrespeito	Autoestima negativa/ Autoestima Positiva	Relato de autodepreciação	0,75 (0,70–0,79)	0,86 (0,81–0,89)
		Sentimento de inutilidade	0,89 (0,87–0,92)	0,88 (0,86–0,91)
Liberdade e participação	Negação do tratamento/Aceitação do tratamento	Recusa verbal de adesão ao regime terapêutico	0,78 (0,73–0,82)	0,85 (0,80–0,88)
		Justificativas de não aceitação da condição clínica	0,97 (0,95–1,00)	0,87 (0,85–0,91)
Educação para saúde	Letramento em saúde, Prejudicado/Letramento em saúde, Adequado	Dificuldade de compreender prescrições médicas	0,79 (0,74–0,83)	0,86 (0,81–0,89)
		Incapacidade de seguir orientações de autocuidado	0,93 (0,88–0,96)	0,83 (0,80–0,86)
Autorrealização	Capacidade laboral, Diminuída/Capacidade laboral, Melhorada	Relato de afastamento do trabalho por limitações físicas ou emocionais	0,74 (0,69–0,78)	0,86 (0,81–0,89)
		Incapacidade de realizar tarefas ocupacionais habituais	0,93 (0,89–0,96)	0,95 (0,93–0,98)
Religiosidade e espiritualidade	Sofrimento espiritual/Sofrimento, Ausente	Angústia	0,73 (0,68–0,77)	0,86 (0,81–0,89)
		Sentimento de abandono por divindade ou comunidade de fé	0,86 (0,82–0,88)	0,86 (0,81–0,90)

Legenda: Se – sensibilidade; Es – especificidade; IC 95% – intervalo de confiança de 95%.

A análise dos resultados evidencia que o estudo alcançou excelente desempenho na validação clínica das intervenções, conforme apresentado na Tabela 3. As três classes latentes apresentaram valores de sensibilidade e especificidade elevados, todos acima de 80%, com intervalos de confiança estreitos, o que reforça a robustez estatística e a consistência dos achados.

**Tabela 3:** Exemplos de IE agrupadas por classe latente, com medidas de desempenho diagnóstico - Rio de Janeiro, RJ, Brasil, 2025.

Classe latente	Perfil clínico predominante	Intervenções Prioritárias	S (%) IC95%	E (%) IC95%	F (%)
Classe 1	Baixo risco	Monitorar sinais vitais; Orientar dieta equilibrada; Promover adesão ao tratamento; Estimular vínculo familiar; Reforçar cuidados com a pele; Avaliar repouso e qualidade do sono;	82,5 (79,0 – 85,6)	87,2 (84,0 – 90,1)	36,8%
Classe 2	Médio risco	Incentivar adesão ao tratamento como forma de redução de sofrimento futuro; Avaliar dor e náuseas; Estimular autocuidado; Promover expressão emocional; Incentivar autoestima, autoconfiança e aceitação da doença; Oferecer conforto físico e psicológico;	85,3 (82,1 – 88,0)	89,0 (86,2 – 91,5)	41,5%
Classe 3	Alto risco	Gerenciar intercorrências respiratórias e hemodinâmicas; Regular diurese, glicemia e PA; Intensificar vigilância neurológica; Atuar no sofrimento espiritual; Reforçar importância da hemodiálise regular para reduzir risco de mortalidade.	90,1 (87,5 – 92,4)	92,0 (89,8 – 94,0)	21,7%

Legenda: Se – sensibilidade; Es – especificidade; IC 95% – intervalo de confiança de 95%.

A Classe 1 concentrou a maior frequência da amostra (36,8%), refletindo o perfil predominante de pacientes em seguimento regular e com boa adesão terapêutica. A Classe 2 representou 41,5% da população estudada, confirmando a relevância das intervenções educativas e de autocuidado para esse grupo. Já a Classe 3, embora menos prevalente (21,7%), apresentou os maiores índices de acurácia, indicando a precisão do modelo em identificar casos complexos e de maior gravidade.

Esse desempenho global sugere que o instrumento utilizado possui elevada aplicabilidade clínica, contribuindo para maior resolutividade no cuidado de pessoas em hemodiálise. Em razão do número limitado de páginas, a Tabela 3 apresenta exemplos de IE agrupadas por classe latente, com suas respectivas medidas de desempenho diagnóstico.

## DISCUSSÃO

Os achados deste estudo demonstram que a aplicação da ACL com efeitos aleatórios foi eficaz na validação clínica dos indicadores da CIPE^®^, permitindo a identificação de padrões diagnósticos com elevada sensibilidade, especificidade e acurácia global. Essa abordagem estatística reforça a confiabilidade dos indicadores clínicos observáveis e sua capacidade de discriminar perfis latentes distintos, contribuindo diretamente para a qualificação da prática diagnóstica em enfermagem.

Esses resultados estão alinhados com estudo que mapeou evidências sobre a validação clínica de subconjuntos terminológicos da CIPE^®^ e identificou que, embora a maioria das pesquisas ainda esteja em fase inicial, há crescente reconhecimento da importância da validação clínica como etapa essencial para consolidar a linguagem padronizada na prática assistencial(^1)^.

Além disso, outro estudo destaca que a validação clínica contribui para a melhoria da qualidade do cuidado, ao permitir que os termos utilizados reflitam com maior precisão as necessidades reais da clientela^(12)^. No presente estudo, a associação entre indicadores e classes latentes possibilitou não apenas validar os termos, mas também agrupar intervenções específicas por perfil clínico, representando um avanço metodológico em relação a investigações anteriores, que se concentraram majoritariamente na validação de conteúdo ou semântica.

No domínio das necessidades psicobiológicas, os enunciados de DE/RE e IE apresentaram elevada consistência clínica e robustez, conforme evidenciado pelos altos índices de sensibilidade, especificidade e intervalos de confiança estreitos. Tais achados estão em consonância com estudo metodológico que validou o subconjunto CIPE^®^ para pessoas em hemodiálise, no qual a maioria dos DE/RE e IE concentrou-se nas necessidades psicobiológicas, representando mais de 70% dos enunciados validados^(5)^.

Resultados semelhantes foram observados em pesquisas voltadas à validação de intervenções específicas, como o manejo do excesso de líquidos, que apresentaram altos índices de validação, reforçando a aplicabilidade clínica no cuidado a pacientes em terapia renal substitutiva. Dessa forma, a predominância das necessidades psicobiológicas nos achados desta investigação confirma a centralidade dos aspectos fisiológicos no processo de cuidado a pacientes em hemodiálise, determinantes para a manutenção da segurança clínica e da adesão ao tratamento^(13)^.

Ainda nesse domínio, destacaram-se diagnósticos que traduzem complicações respiratórias, metabólicas e cardiovasculares, como “Dispneia” e “Desequilíbrio de eletrólitos”, cuja validação reforça a importância do monitoramento contínuo e de intervenções direcionadas à estabilidade clínica. Outros enunciados, como “Ingestão Nutricional, Prejudicada”, “Constipação” e “Sono, Inadequado”, evidenciam a relevância de cuidados voltados às dimensões nutricional, gastrointestinal e do repouso, frequentemente comprometidas em virtude das restrições alimentares, da uremia e de sintomas associados. Também se destacam “Fadiga”, “Risco de Infecção”, “Integridade da pele, Prejudicada”, “Hipertensão Arterial”, “Anemia Crônica” e “Febre”, que representam condições de alta prevalência em hemodiálise e demandam estratégias de prevenção, educação em saúde e adesão ao tratamento^(14)^.

O diagnóstico “Memória, Prejudicada” pode refletir alterações cognitivas decorrentes da cronicidade e impactar a adesão às orientações, exigindo maior suporte educativo. Também se destacou o “Desempenho Sexual, Prejudicado”, frequentemente negligenciado na prática clínica, mas que interfere na autoestima, nos relacionamentos interpessoais e na qualidade de vida. Por fim, o diagnóstico “Não adesão ao tratamento hemodialítico”, cuja compreensão ultrapassa a mera frequência às sessões, envolvendo aceitação, motivação e enfrentamento da condição crônica. O reconhecimento desses aspectos amplia a perspectiva de cuidado e reforça a integralidade da assistência de enfermagem às pessoas em hemodiálise^(15-17)^.

No âmbito das necessidades psicossociais, verificou-se a validação de enunciados com consistência clínica moderada, embora menos prevalentes em relação aos psicobiológicos. Esse achado aproxima-se de outros estudos que destacaram a presença relevante, ainda que minoritária, de DE/RE vinculados às necessidades psicossociais, representando aproximadamente 20% dos enunciados validados em subconjuntos terminológicos^(5)^.

Destacaram-se diagnósticos como “Processo Familiar, Prejudicado”, “Lazer, Prejudicado” e “Conforto, Prejudicado”, que refletem as limitações impostas pela rotina dialítica e pelos sintomas associados, comprometendo o convívio familiar, o bem-estar e a qualidade de vida. Somam-se a esses “Autoestima Negativa” e “Negação do Tratamento”, que revelam dificuldades emocionais e de aceitação da cronicidade, bem como “Letramento em Saúde, Prejudicado” e “Capacidade Laboral, Diminuída”, que evidenciam repercussões sociais e educacionais da doença, com impactos diretos na autonomia do autocuidado e na produtividade. Esses achados reforçam a necessidade de intervenções educativas acessíveis, suporte psicológico e estratégias que favoreçam a reorganização do cotidiano e a reinserção social.

Pacientes em hemodiálise frequentemente vivenciam impactos emocionais, como ansiedade, fadiga e comprometimento da autoimagem, elementos que influenciam diretamente sua qualidade de vida. A análise de agrupamentos latentes aplicada a doenças crônicas mostrou que subgrupos de pacientes podem ser diferenciados pela severidade dos sintomas psicossociais, indicando a importância de intervenções direcionadas também a essas necessidades^(18)^.

Assim, a inclusão de enunciados psicossociais validados amplia a perspectiva do cuidado, indo além da dimensão fisiológica e fortalecendo a integralidade da assistência^(19)^. Embora em número reduzido, a validação de enunciados relacionados às necessidades psicoespirituais representa um avanço no reconhecimento da dimensão espiritual no cuidado à pessoa em hemodiálise^(20)^.

No âmbito psicoespiritual, o diagnóstico “Sofrimento Espiritual” evidenciou o peso da dimensão existencial no enfrentamento da doença renal crônica. A dependência de uma terapia contínua e invasiva pode intensificar sentimentos de vazio, perda de sentido e vulnerabilidade, dificultando a adesão e o enfrentamento. A validação desse enunciado aponta para a necessidade de práticas de enfermagem que incorporem a espiritualidade no cuidado, valorizando crenças, valores e recursos internos do paciente como estratégias de fortalecimento da resiliência e promoção do cuidado integral e humanizado.

Em estudo de validação clínica com pacientes em tratamento conservador, diagnósticos como “Sofrimento Espiritual” e “Vínculo Religioso, Prejudicado” foram confirmados como relevantes, mostrando que a dimensão espiritual exerce papel importante no enfrentamento da doença renal crônica^(16)^. Mesmo que a proporção desses enunciados seja pequena, sua incorporação ao plano assistencial revela sensibilidade da CIPE^®^ para abranger o cuidado integral e humanizado, contemplando a experiência existencial dos pacientes^(5,8,21)^.

Os achados deste estudo apresentam convergências e contrastes relevantes quando comparados à validação clínica de subconjuntos terminológicos da CIPE^®^ voltados para pessoas com DRC em tratamento conservador. A primeira diz respeito à predominância de DE/RE e IE relacionados à adesão ao tratamento, ao controle da pressão arterial e ao manejo nutricional. Embora esses elementos também estejam presentes no cuidado de pacientes em hemodiálise, o presente estudo evidenciou especificidades adicionais, como o sofrimento espiritual, a segurança emocional e a sobrecarga clínica decorrente das intercorrências dialíticas. Além disso, a estratificação dos participantes por classes latentes permitiu identificar padrões assistenciais distintos, o que não foi observado nos estudos com pacientes em tratamento conservador. Esses contrastes reforçam a importância de adaptar os subconjuntos terminológicos às realidades clínicas específicas, garantindo maior precisão na identificação das NHB e na personalização das intervenções de enfermagem^(9)^.

Portanto, os resultados obtidos neste estudo não apenas validam clinicamente os indicadores da CIPE^®^, como também ampliam sua aplicabilidade ao propor uma estratégia de estratificação diagnóstica e de intervenção por perfil clínico. Tal proposta representa uma contribuição inovadora para a prática baseada em evidências e para o fortalecimento da linguagem profissional da enfermagem.

A presente análise demonstrou a aplicabilidade da Teoria das NHB, aliada à ACL, como método robusto para a identificação de padrões diagnósticos em pacientes em tratamento hemodialítico. A adoção de um modelo com três classes latentes possibilitou a estratificação clínica dos pacientes em níveis distintos de complexidade, oferecendo subsídios estatísticos relevantes para o planejamento personalizado do cuidado.

As elevadas medidas de sensibilidade e especificidade observadas em diversos indicadores clínicos reforçam sua capacidade discriminativa. Os indicadores associados à oxigenação, regulação vascular, nutrição e eliminação apresentaram maior consistência na Classe 3, evidenciando que aspectos fisiológicos críticos tendem a concentrar-se em pacientes com maior comprometimento assistencial. Por outro lado, os indicadores dos domínios psicossociais, emocionais e espirituais mostraram desempenho adequado, especialmente na Classe 2, demonstrando que tais manifestações não são exclusivas de quadros graves e requerem atenção transversal na prática de enfermagem.

O agrupamento de intervenções por classe latente contribui diretamente para a prática assistencial, ao permitir a associação entre perfil clínico e plano de cuidado individualizado. A composição das intervenções por classe sugere que pacientes da Classe 1 se beneficiam de ações educativas e preventivas; os da Classe 2 demandam intervenções de apoio emocional, controle sintomático e fortalecimento do autocuidado; e os da Classe 3 exigem planos complexos e interdisciplinares para manejo intensivo de complicações clínicas e sofrimento psicossocial.

Assim, os resultados reforçam o potencial da abordagem multidimensional na construção de DE/RE e IE, evidenciam a capacidade da ACL em gerar conhecimento clínico aplicável e apontam caminhos promissores para a consolidação da prática baseada em evidências dentro dos sistemas classificatórios da CIPE^®^.

No que diz respeito às limitações do estudo, a seleção de uma população específica pode ter reduzido a abrangência dos achados, tornando sua generalização mais desafiadora. Além disso, o fato de o estudo estar restrito a uma única região e contexto introduziu uma característica cultural singular, que deve ser considerada atentamente na análise dos resultados, garantindo maior precisão na interpretação dos achados.

A implementação do subconjunto terminológico validado para pessoas em hemodiálise exige uma abordagem estruturada e integrada à rotina dos profissionais de enfermagem. Primeiramente, é fundamental promover treinamentos contínuos para capacitar enfermeiros na correta utilização dos enunciados de DE/RE e IE da CIPE^®^. A realização de workshops e simulações clínicas pode contribuir para consolidar o raciocínio clínico necessário à identificação dos indicadores específicos dos diagnósticos desse subconjunto e para a tomada de decisões assertivas.

Outro aspecto essencial para a implementação bem-sucedida do subconjunto terminológico é a criação de protocolos assistenciais baseados nas definições validadas. A incorporação desses termos nos manuais de conduta em hemodiálise pode garantir maior uniformidade nas práticas de enfermagem e reduzir variações na condução dos casos clínicos. Além disso, os critérios de validação podem servir como base para auditorias internas, permitindo monitorar a adesão às melhores práticas e identificar oportunidades de melhoria no cuidado prestado.

Por fim, a realização de estudos multicêntricos constitui um passo estratégico para ampliar a aplicabilidade do subconjunto validado em diferentes instituições de saúde. A replicação do modelo assistencial em outros serviços de hemodiálise permitirá avaliar sua eficácia em diversos contextos, reforçando sua utilidade na prática clínica. Para garantir a continuidade da atualização, recomenda-se que os profissionais envolvidos participem de redes de colaboração científica, onde possam compartilhar achados e contribuir para a evolução do subconjunto terminológico da CIPE^®^.

A implementação eficaz desse subconjunto representa um avanço significativo na padronização da linguagem especializada da enfermagem em hemodiálise, fortalecendo a tomada de decisão dos profissionais, aprimorando a segurança do paciente e garantindo uma assistência mais qualificada aos usuários.

## CONCLUSÃO

A pesquisa evidenciou a validação clínica de um subconjunto composto por 23 DE/RE e 169 IE da CIPE^®^, direcionado ao cuidado de pessoas com DRC em tratamento hemodialítico. Os achados deste estudo demonstram que a aplicação da ACL constitui uma abordagem metodológica robusta para a validação clínica de subconjuntos terminológicos da CIPE^®^, especialmente em contextos marcados pela complexidade assistencial, como o cuidado ao paciente em hemodiálise.

A identificação de três classes latentes permitiu diferenciar perfis clínicos distintos, com elevados níveis de sensibilidade, especificidade e acurácia global, reforçando a validade dos indicadores observáveis para fins diagnósticos. Com isso, este estudo contribui para a consolidação da linguagem padronizada da enfermagem, fortalece a prática baseada em evidências e oferece subsídios para a construção de protocolos assistenciais mais precisos, seguros e individualizados.

A aplicabilidade prática do subconjunto é ampliada ao possibilitar a identificação precoce de riscos, o planejamento de intervenções específicas e o monitoramento contínuo dos resultados de enfermagem. Recomenda-se que futuras pesquisas explorem sua implementação em diferentes contextos assistenciais, como a atenção primária, unidades de terapia intensiva e serviços de transplante renal, além de investigar seu impacto na qualidade do cuidado, nos desfechos clínicos e na gestão dos serviços de saúde.

## Data Availability

Todo o conjunto de dados que dá suporte aos resultados deste estudo está disponível mediante solicitação ao autor correspondente.

# Translation: Validación clínica del Subconjunto Terminológico del CIPE^®^ para personas con enfermedad renal crónica en hemodiálisis

**DOI:** 10.1590/1980-220X-REEUSP-2025-0364es

**Authors:** Richardson Augusto Rosendo da Silva, Juliana Otaciana dos Santos, Silvia Maria de Sá Basílio Lins, Harlon França de Menezes, Ana Beatriz Pereira da Silva, José Leonildo Fernandes de Queiroz, Rafaella Viesti Machado, Jéssica Fernanda Galon

**Affiliations:** 1 Universidade Federal do Rio Grande do Norte, Programa de Pós-graduação em Enfermagem, Natal, RN, Brasil.; 2 Universidade Federal Fluminense, Programa de Pós-graduação Mestrado Profissional em Enfermagem Assistencial, Niterói, RJ, Brasil.; 3 Universidade Federal do Rio Grande do Norte, Departamento de Enfermagem, Natal, RN, Brasil.

**Keywords:** Investigación en Enfermería Clínica, Estudio de Validación, Terminología Normalizada de Enfermería, Diálisis Renal, Insuficiencia Renal Crónica

## Abstract

**Objetivo:**

Validar clínicamente las declaraciones de diagnóstico/resultados y las intervenciones de enfermería del subconjunto CIPE^®^ para personas con enfermedad renal crónica sometidas a hemodiálisis.

**Método:**

Estudio de validación clínica, con individuos de 18 años o más, con enfermedad renal crónica, sometidos a tratamiento de diálisis durante al menos tres meses. La investigación se llevó a cabo en tres etapas: Aplicación del proceso de enfermería a la práctica clínica, que comprende cinco pasos esenciales – evaluación, diagnóstico, planificación, implementación y evaluación de Enfermería; Estructuración de estudios de caso; e Identificación de la prevalencia de diagnósticos/resultados de enfermería e intervención de enfermería enumerados en los estudios de caso. Los datos se analizaron mediante análisis de clases latentes.

**Resultados:**

Los hallazgos revelaron tres clases latentes distintas: riesgo bajo (Clase 1), riesgo medio (Clase 2) y riesgo alto (Clase 3). Los indicadores mostraron altos valores de sensibilidad (72,0% a 91,0%) y especificidad (83,0% a 92,0%), lo que confirma la validez clínica del subconjunto y la consistencia del modelo para discriminar diferentes perfiles de pacientes de hemodiálisis.

**Conclusión:**

El estudio validó clínicamente un subconjunto de 23 diagnósticos/resultados de enfermería y 169 intervenciones de enfermería, demostrando su aplicabilidad en la práctica clínica.

## INTRODUCCIÓN

La Clasificación Internacional para la Práctica de Enfermería (CIPE^®^) desempeña un papel esencial en la documentación de las acciones del enfermero en la práctica clínica, garantizando la estandarización, la organización y una mayor precisión en los registros asistenciales. Este recurso favorece una comunicación clara y eficaz entre los profesionales de la salud, además de facilitar la documentación de la asistencia prestada^(1)^. La CIPE^®^ permite el desarrollo de subconjuntos terminológicos, que reúnen enunciados de diagnósticos/resultados (DE/RE) e intervenciones de enfermería (IE), dirigidos a grupos específicos de pacientes^(2)^.

Así, se puede definir la CIPE^®^ como un sistema de comunicación especializado y representativo de la práctica de enfermería, con alcance local y global, que contribuye a la aplicación eficaz del Proceso de Enfermería (PE). Este lenguaje constituye una estrategia eficiente para la Recolección, el almacenamiento y el análisis de datos en enfermería, lo que favorece el fortalecimiento y la valorización de la profesión. Cabe destacar que, para mejorar la estructuración de los Subconjuntos Terminológicos y aumentar su eficacia, es esencial validarlos en el contexto clínico^(1)^.

Para una mejor estructuración de esta tecnología y para hacerla más eficaz, es fundamental la aplicación de un modelo teórico o conceptual que sirva de base a la práctica clínica. Entre los principales modelos teóricos, destaca el de las Necesidades Humanas Básicas (NHB), que constituye la base de la práctica de enfermería, permitiendo comprender al ser humano de forma integral y multidimensional. Propuestas originalmente por Wanda Horta, estas necesidades se clasifican en psicobiológicas, psicosociales y psicoespirituales, reflejando las dimensiones física, relacional y existencial de la atención. En el contexto de la Enfermedad Renal Crónica (ERC) y la terapia hemodialítica, las NHB se ven a menudo comprometidas, lo que exige un enfoque asistencial centrado en el individuo^(3)^.

Según datos del último boletín epidemiológico sobre ERC en Brasil, se estima que alrededor del 10 % de la población presenta algún grado de compromiso renal. En 2023, se registraron más de 187 000 atendimientos en Atención Primaria de Salud relacionados con la ERC, con predominio entre personas de 50 a 79 años^(1-3)^. El aumento significativo de las hospitalizaciones por esta afección, de 84 337 en 2010 a 140 648 en 2023, pone de manifiesto la sobrecarga de los servicios de salud y la complejidad clínica de los pacientes en hemodiálisis^(4)^. Este escenario refuerza la necesidad de intervenciones basadas en modelos que consideren la integralidad del ser humano, como el de las NHB, y de herramientas que estandaricen el lenguaje profesional, como los Subconjuntos Terminológicos de la CIPE^®^.

Estudios previos ya han llevado a cabo la construcción de la terminología y la validación del contenido de un subconjunto terminológico de la CIPE^®^ para personas con enfermedad renal crónica en hemodiálisis, a la luz del modelo de las NHB. Su elaboración surgió de la necesidad de estandarizar los registros de la práctica de enfermería y mejorar la calidad de la documentación asistencial en los servicios de hemodiálisis^(5,6)^.

En este sentido, el método brasileño fomenta la validación clínica de los subconjuntos, con el fin de garantizar su aplicabilidad y confiabilidad en la práctica asistencial. La validación clínica permite verificar si las afirmaciones desarrolladas reflejan adecuadamente las condiciones reales de las personas con ERC en hemodiálisis, asegurando que los DE/RE y los IE sean eficaces en el contexto clínico. Además, este proceso permite realizar los ajustes necesarios, mejorando la precisión de la terminología utilizada y favoreciendo una atención más dirigida y segura^(7)^.

El estado actual del uso de la CIPE^®^ en la atención a personas en hemodiálisis demuestra que aún existe una brecha en cuanto a la validación clínica de subconjuntos terminológicos específicos para esta población. La enfermería en nefrología exige instrumentos estandarizados que permitan una comunicación eficaz entre los profesionales, además de mejorar la precisión diagnóstica y la implementación de intervenciones específicas. Sin embargo, se observa una ausencia de estudios que evalúen la aplicabilidad y la confiabilidad de los enunciados del CIPE^®^ en personas con ERC en hemodiálisis, lo que dificulta su integración en la práctica clínica y en la documentación sistematizada de la atención^(8-10)^.

Ante este vacío de conocimiento, esta investigación busca llenar dicha necesidad al validar clínicamente un subconjunto terminológico para personas con enfermedad renal crónica en hemodiálisis, ofreciendo una base científica que fortalezca la toma de decisiones de los profesionales y contribuya a la seguridad y calidad de la atención prestada, lo que justifica la realización del presente estudio.

La relevancia clínica del estudio radica en ofrecer importantes aportes para la atención de enfermería a pacientes renales hospitalizados, al proponer un conjunto de términos que reflejen con mayor fidelidad las necesidades clínicas de estos pacientes. Además, la validación clínica fortalece la práctica basada en la evidencia y puede respaldar los protocolos de atención, los planes de cuidados y la enseñanza de enfermería. Por otra parte, promueve la valorización del lenguaje profesional y la visibilidad de las acciones de enfermería. Por lo tanto, este estudio tiene como objetivo validar clínicamente los enunciados de diagnósticos/resultados e intervenciones de enfermería del Subconjunto CIPE^®^ para personas con enfermedad renal crónica en hemodiálisis.

## MÉTODO

### Diseño y Lugar del Estudio

Se trata de un estudio de validación clínica, utilizando el Análisis de Clases Latentes (ACL)^(11)^, en el que se aplicaron estudios de casos realizados de acuerdo con las fases establecidas por el Proceso de Enfermería (PE). La investigación se llevó a cabo en un consultorio de enfermedades renales situado en un municipio de la región noreste de Brasil. Además, el estudio se basó en la iniciativa *Standards for Reporting Studies of Diagnostic Accuracy* (STARD) y en pruebas diagnósticas, con el objetivo de establecer la precisión de los indicadores^(1)^.

Como base para la validación clínica, se utilizó el subconjunto terminológico previamente validado en relación con el contenido en un estudio anterior, compuesto por 81 enunciados de DE, 242 enunciados de RE y 533 enunciados de IE, a la luz de la Teoría de las NHB^(5)^.

### Población, Criterios de Selección y Definición de la Muestra

El tamaño de la muestra siguió las recomendaciones del modelo de clase latente, que prevé una relación de 5 a 30 participantes por indicador evaluado^(11)^. En el presente estudio, se adoptó la proporción de cinco pacientes para cada uno de los 46 indicadores evaluados, obteniéndose una muestra final de 230 participantes.

La muestra estuvo compuesta por personas con enfermedad renal crónica, mayores de 18 años y en tratamiento dialítico en el servicio durante al menos tres meses, según los criterios de inclusión establecidos. Se excluyó a los pacientes sin capacidad cognitiva para participar en la investigación, verificada a partir de la información registrada en los expedientes médicos, tales como: evoluciones médicas y de enfermería que describían cuadros de demencia, trastornos neuropsiquiátricos o compromiso cognitivo significativo; informes de evaluación neuropsicológica o psiquiátrica; y prescripción de medicamentos indicativos de trastornos cognitivos graves.

La selección de los participantes se realizó de forma intencionada, basándose en la revisión de los expedientes médicos y en la evaluación clínica previa realizada por el investigador responsable. Se invitó a los pacientes a realizar pequeñas tareas cognitivas, como informar su nombre completo, fecha de nacimiento y reconocer el lugar y el momento de la entrevista. El objetivo era garantizar que todos los participantes tuvieran plena capacidad de comunicación, comprensión y consentimiento, tal y como se exige para la aplicación del Proceso de Enfermería y la Recolección de datos.

### Recolección de Datos

Para alcanzar el objetivo establecido, el estudio se estructuró y llevó a cabo en tres fases distintas: 1) Aplicación del Proceso de Enfermería a la práctica clínica, que abarca cinco etapas esenciales: evaluación, diagnóstico, planificación, implementación y evolución de la enfermería; 2) Estructuración de los estudios de caso; 3º) Identificación de la prevalencia de los DE/RE e IE enumerados a partir de los estudios de caso. El estudio se llevó a cabo entre agosto de 2024 y julio de 2025.

Para orientar la recopilación de datos, se estableció un protocolo que preveía el seguimiento de los participantes en dos etapas: la primera consistía en una consulta de enfermería, seguida de una segunda consulta destinada a la evaluación. No se definió un intervalo mínimo entre las consultas, ya que el protocolo priorizó la flexibilidad en el seguimiento, respetando la disponibilidad individual y las demandas específicas de cada caso. Este enfoque tenía como objetivo garantizar una mayor adhesión al estudio y minimizar posibles pérdidas de muestras, sin comprometer la calidad de los datos recopilados. Esta etapa se llevó a cabo entre agosto de 2024 y febrero de 2025.

### Primera Etapa

En la primera fase, la Recolección de datos, el examen físico y los registros fueron realizados por el investigador principal y siete estudiantes de posgrado, con el apoyo de un instrumento basado en la Teoría de las NHB, lo que garantizó un enfoque estructurado y fundamentado.

Los siete estudiantes de posgrado involucrados eran enfermeros con experiencia en el uso del CIPE^®^ y el PE, y recibieron una formación específica e intensiva impartida por el investigador principal. Esta formación incluyó una revisión teórica sobre las NHB, la aplicación práctica del instrumento de recopilación y simulaciones clínicas centradas en la identificación de DE/RE e IE según el CIPE^®^.

La preparación tenía como objetivo garantizar la uniformidad en la aplicación del instrumento, la fidelidad conceptual al modelo teórico adoptado y la calidad de los registros clínicos, asegurando que los datos reflejaran con precisión la realidad asistencial de los pacientes en hemodiálisis. El instrumento de recolección se estructuró en secciones que incluían datos sociodemográficos, evaluación, diagnóstico, planificación, implementación y evolución de la enfermería.

Además, se analizaron exámenes de laboratorio y de imagen para complementar la evaluación clínica.

Inicialmente, se realizó una prueba piloto, previamente aprobada por el Comité de Ética en Investigación, con el objetivo de verificar la idoneidad del instrumento. Los participantes en esta prueba no se incluyeron en la muestra final, lo que garantizó la integridad de los datos recopilados.

Los enunciados de DE/RE se establecieron sobre la base de indicadores clínicos descritos en las definiciones operativas del subconjunto terminológico de CIPE^®^. Para su organización y accesibilidad, estas declaraciones se sistematizaron en un formulario electrónico en Google Docs.

Las etapas de planificación e implementación se llevaron a cabo de acuerdo con los RE establecidos para cada DE, según lo definido por el subconjunto terminológico. Las IE propuestas se orientaron para satisfacer las necesidades identificadas, asegurando un enfoque personalizado y alineado con los objetivos de la atención. La etapa de evaluación se llevó a cabo centrándose en los enunciados de DE previamente identificados, lo que permitió verificar la eficacia de las intervenciones y la evolución clínica de los participantes.

### Segunda Etapa

En la segunda fase, los investigadores desarrollaron los estudios de caso, formularon las declaraciones de DE/RE e IE e identificaron su prevalencia. Esta información se organizó en una hoja de cálculo de Microsoft Excel^®^, estructurada en pestañas separadas, cada una correspondiente a un paciente. Posteriormente, las declaraciones se clasificaron según las necesidades psicobiológicas, psicoespirituales y psicosociales.

### Tercera Etapa

En la fase final, se empleó el análisis de clases latentes para la validación clínica del subconjunto terminológico, basándose en pacientes con DRC en hemodiálisis, con el fin de verificar los patrones clínicos relevantes que sustentaran el subconjunto del CIPE^®^. Durante esta etapa, se consideraron clínicamente validados los DE/RE e IE identificados en los pacientes, tal y como se describen en los estudios de caso, que se incorporaron al subconjunto terminológico.

### Análisis y Tratamiento de los Datos

El análisis descriptivo incluyó el cálculo de frecuencias absolutas y porcentuales de los enunciados de DE/RE, además de la prueba de Shapiro-Wilk. Debido al gran número de enunciados, los IE se agruparon según la categoría del modelo teórico y se discutieron en función de la frecuencia.

Para garantizar la precisión en la organización de los datos, toda la información se registró en una hoja de cálculo electrónica. En el contexto de esta investigación, se consideraron clínicamente validados los enunciados de DE/RE e IE identificados en los pacientes, documentados en los estudios de caso y presentes en el subconjunto terminológico.

Los indicadores clínicos se midieron mediante entrevistas y exámenes físicos. Los datos se analizaron con la ayuda de los programas *Statistical Package for the Social Sciences* (SPSS) y R (v. 2.12.1). Se utilizó el método de análisis de clases latentes para calcular la sensibilidad y la especificidad de los indicadores. El indicador se consideró estadísticamente significativo si al menos uno de sus intervalos de confianza era >0,5.

Las variables se codificaron de forma binaria (presencia = 1; ausencia = 0). El modelado se realizó con el software R, utilizando el paquete *poLCA*. Se probaron modelos con 2 a 5 clases, siendo el modelo con tres clases el más adecuado, según el menor valor del BIC (*Bayesian Information Criterion*) y la entropía ≥ 0,80.

Los perfiles latentes identificados se interpretaron en función de la frecuencia condicional de los DE/RE, lo que permitió reconocer patrones clínicos distintos, descritos en la sección de Resultados. El ACL identificó tres grupos clínicos con perfiles distintos en cuanto a la expresión de los DE/RE de enfermería: Clase 1 (bajo riesgo): pacientes clínicamente compensados, con alta adherencia al tratamiento y ausencia de síntomas físicos o emocionales significativos; Clase 2 (riesgo medio): baja manifestación de signos físicos, pero fuerte prevalencia de DE de naturaleza psicosocial que podrían interferir en la adherencia al tratamiento; Clase 3 (alto riesgo): pacientes críticos multifactoriales, con alta frecuencia de síntomas físicos, baja adherencia al tratamiento, además de sufrimiento espiritual y daños en los lazos religiosos.

Las clases se definieron a partir de la presencia de combinaciones específicas de signos y síntomas/indicadores clínicos. La sensibilidad indicó la capacidad del modelo para identificar correctamente a los pacientes pertenecientes a cada clase, mientras que la especificidad evaluó en qué medida el modelo evitó clasificar como pertenecientes a la clase a individuos que no encajaban en ella.

### Aspectos Éticos

El presente estudio se llevó a cabo de conformidad con los principios éticos de voluntariedad, anonimato y confidencialidad, cumpliendo con las directrices de la Resolución n.º 466/12 del Consejo Nacional de Salud, que regula las investigaciones con seres humanos. La investigación fue aprobada por el Comité de Ética en Investigación de una institución federal de educación superior, de conformidad con el dictamen n.º 7.753.717. Para garantizar la participación voluntaria e informada, todos los participantes firmaron el Término de Consentimiento Libre e Informado (TCLI), garantizando el cumplimiento de las normas éticas y científicas aplicables.

## RESULTADOS

La muestra estuvo compuesta por 230 individuos en tratamiento hemodialítico debido a una enfermedad renal crónica, distribuidos entre ambos sexos, siendo el 41 % mujeres y el 59 % hombres. La mayoría tenía 60 años o más (86 %), mientras que solo el 14 % tenía menos de 60 años. En cuanto al nivel educativo, predominó la educación secundaria incompleta (58 %). En cuanto al estado civil, el 69 % estaba casado y, en cuanto a la condición socioeconómica, el 44 % declaró tener ingresos iguales o superiores a dos salarios mínimos. Se observó que todos tenían alguna comorbilidad asociada, siendo la hipertensión arterial la más prevalente (91%), seguida de la diabetes mellitus (81%).

En lo que respecta al subconjunto original, se implementaron 23 enunciados de DE/RE y 169 IE, con un promedio de cinco enunciados de DE/RE por estudio de caso, variando entre un mínimo de dos y un máximo de 12.

La Tabla 1 presenta las medidas de precisión diagnóstica para cada indicador clínico, organizadas según las necesidades psicobiológicas humanas. Se incluyen: Sensibilidad (Se%) y sus respectivos intervalos de confianza del 95% (IC95%), así como Especificidad (Es%) con sus IC95%. Estos valores demuestran que el modelo de Análisis de Clases Latentes generó estimaciones robustas para la validación clínica de estos indicadores.

**Tabla 1:** Frecuencia y medidas de precisión diagnóstica de los indicadores clínicos por necesidades psicobiológicas humanas - Río de Janeiro, RJ, Brasil, 2025.

Necesidad humana	Diagnóstico/ resultado de enfermería	Indicador clínico	S (%) IC95%	E (%) IC95%
Oxigenación	Disnea/Disnea, Ausente	Frecuencia respiratoria ≥ 20 irpm	0,94 (0,91–0,96)	0,88 (0,83–0,91)
		Saturación periférica de O_2_ < 92%	0,88 (0,84–0,91)	0,91 (0,87–0,94)
Hidratación	Desequilibrio de electrólitos/Equilibrio de electrólitos, Adecuado	Niveles séricos alterados de sodio, potasio y calcio	0,86 (0,82–0,88)	0,89 (0,86–0.91)
		Presencia de calambres o debilidad muscular	0,85 (0,80–0,88)	0,90 (0,86–0,93)
Nutrición	Ingesta Nutricional, Perjudicada/Ingesta Nutricional, Mejorada	Índice de masa corporal (IMC) fuera de límites adecuados	0,90 (0,86–0,94)	0,95 (0,93–0,98)
		Exámenes de laboratorio alterados (albúmina, proteínas totales)	0,80 (0,75–0,84)	0,87 (0,82–0,90)
Eliminación	Constipación/Constipación, Mejorada	Evacuación ausente por más de 72 horas	0,94 (0,90–0,99)	0,91 (0,88–0,95)
		Heces endurecidas y resecas	0,93 (0,90–0,97)	0,85 (0,82–0,88)
Sueño y reposo	Sueño, Inadecuado/Sueño, Adecuado	Relato de dificultad para iniciar el sueño	0.90 (0.87–0.95)	0.94 (0.90–0.97)
		Uso de medicación inductora de sueño	0,82 (0,77–0,86)	0,86 (0,81–0,89)
Actividad física	Cansancio/Cansancio, Ausente	Relato de cansancio desproporcional al esfuerzo	0,87 (0,85–0,89)	0,94 (0,91–0,97)
		Necesidad de reposo frecuente	0,83 (0,78–0,87)	0,88 (0,84–0,91)
Sexualidad y reproducción	Desempeño sexual, Perjudicado/Desempeño sexual, Mejorado	Relato de disfunción eréctil o anorgasmia	0,76 (0,71–0,80)	0,86 (0,81–0,89)
		Reducción de deseo sexual	0,95 (0,90–0,99)	0,85 (0,81–0,88)
Seguridad física y ambiental	Riesgo de Infección/Risco de Infección, Ausente	Presencia de dispositivo invasivo (catéter, acceso venoso)	0,86 (0,81–0,89)	0,87 (0,82–0,90)
		Integridad cutánea perjudicada	0,90 (0,87–0,93)	0,89 (0,86–0,94)
Integridad física	Integridad de la piel, Perjudicada/Integridad de la piel, Mejorada	Presencia de lesiones o úlceras de presión	0,89 (0,85–0,93)	0,92 (0,90–0,94)
		Retardo en cicatrización de heridas	0,80 (0,75–0,84)	0,86 (0,81–0,89)
Regulación vascular	Hipertensión Arterial/Presión Arterial, en Límites Normales	Presión arterial ≥ 140/90 mmHg en reposo	0,91 (0,87–0,94)	0,86 (0,81–0,89)
		Edema en miembros inferiores	0,85 (0,80–0.87)	0,88 (0,85–0,92)
Regulación térmica	Fiebre/Fiebre, Ausente	Temperatura corporal > 37,8 °C	0,76 (0,71–0,80)	0,84 (0,79–0,88)
		Escalofríos recurrentes	0,86 (0,83–0,90)	0,94 (0,90–0,98)
Regulación neurológica	Memoria, Perjudicada/Memoria, Mejorada	Olvido frecuente de hechos recientes	0,80 (0,75–0,84)	0,85 (0,80–0,88)
		Dificultad en recordar instrucciones simples	0,94 (0.91–0.96)	0,88 (0,83–0,91)
Regulación hormonal	Anemia, Crónica/Anemia, Mejorada	Hemoglobina < 11 g/dL	0,84 (0,79–0,88)	0,88 (0,83–0,91)
		Palidez cutáneo-mucosa	0,93 (0.89–0,96)	0,95 (0,93–0,98)
Senso-percepción	Dolor, Crónico/Dolor Crónico, Ausente	Relato de dolor persistente > 3 meses	0,84 (0,79–0,88)	0,87 (0,82–0,90)
		Uso continuo de analgésicos	0.91 (0,87–0,94)	0,84 (0,80–0,88)
Terapéutica e prevención	No Adhesión al Tratamiento Hemodialítico/Adhesión al Tratamiento Hemodialítico	Ganancia de peso interdialítico en más de lo recomendado	0,87 (0,84–0,90)	0,84 (0,80–0,87)
		Alteraciones de laboratorio por no adhesión (urea, creatinina, potasio)	0,87 (0,83–0,91)	0,91 (0,88–0,94)

Abreviaturas: Se – sensibilidad; Es – especificidad; IC 95% – intervalo de confianza de 95%.

Las medidas de precisión de los indicadores clínicos, referidos a las necesidades psicosociales y psicoespirituales humanas, se presentan en la Tabla 2.

**Tabla 2:** Frecuencia y medidas de precisión diagnóstica de los indicadores clínicos por necesidades psicosociales y psicoespirituales humanas – Río de Janeiro, RJ, Brasil, 2025.

Necesidad humana	Diagnóstico/resultado de enfermería	Indicador clínico	S (%) IC95%	E (%) IC95%
Gregaria (relaciones sociales)	Proceso Familiar, Perjudicado/Proceso Familiar, Adecuado	Aislamiento o alejamiento de la convivencia familiar	0,75 (0.70–0,79)	0,85 (0,80–0,88)
		Falta de apoyo emocional relatada por el paciente	0,97 (0,94–1,00)	0,91 (0,88–0,94)
Recreación e esparcimiento	Esparcimiento perjudicado/Esparcimiento Mejorado	Ausencia de actividades recreativas en la rutina	0,76 (0,71–0,80)	0,86 (0,81–0,89)
		Relato de desinterés por esparcimiento	0,86 (0,84–0,88)	0,90 (0,85–0,94)
Seguridad emocional	Confort perjudicado/Confort Mejorado	Relato de malestar general	0,80 (0,75–0,84)	0,84 (0,79–0,88)
		Expresión facial de dolor o incomodidad	0,95 (0,91–0,97)	0,87 (0,84–0,92)
Autoestima e auto-respeto	Autoestima negativa/Autoestima Positiva	Relato de auto-depreciación	0,75 (0,70–0,79)	0,86 (0,81–0,89)
		Sentimiento de inutilidad	0,89 (0,87–0,92)	0,88 (0,86–0,91)
Libertad y participación	Negación del tratamiento/Aceptación del tratamiento	Recusa verbal de adhesión al régimen terapéutico	0,78 (0,73–0,82)	0,85 (0,80–0,88)
		Justificativas de no aceptación de la condición clínica	0,97 (0,95–1,00)	0,87 (0,85–0,91)
Educación para la salud	Alfabetización en salud, Perjudicado/Alfabetización en salud, Adecuado	Dificultad en comprender prescripciones médicas	0,79 (0,74–0,83)	0,86 (0,81–0,89)
		Incapacidad de seguir orientaciones de autocuidado	0,93 (0,88–0,96)	0,83 (0,80–0,86)
Autorrealización	Capacidad laboral, Disminuida/Capacidad laboral, Mejorada	Relato de ausencia del trabajo por limitaciones fí o emocionales	0,74 (0,69–0,78)	0,86 (0,81–0,89)
		Incapacidad de realizar tareas ocupacionales habituales	0,93 (0,89–0,96)	0,95 (0,93–0,98)
Religiosidad y espiritualidad	Sufrimiento espiritual/Sufrimiento, Ausente	Angustia	0,73 (0,68–0,77)	0,86 (0,81–0,89)
		Sentimiento de abandono por divinidad o comunidad de fe	0,86 (0,82–0,88)	0,86 (0,81–0,90)

Abreviaturas: Se – sensibilidad; Es – especificidad; IC 95% – intervalo de confianza de 95%.

El análisis de los resultados evidencia que el estudio alcanzó un excelente desempeño en la validación clínica de las intervenciones, tal como se presenta en la Tabla 3. Las tres clases latentes presentaron valores elevados de sensibilidad y especificidad, todos superiores al 80 %, con intervalos de confianza estrechos, lo que refuerza la solidez estadística y la consistencia de los hallazgos.

**Tabla 3:** Ejemplos de IE agrupadas por clase latente, con medidas de rendimiento diagnóstico – Río de Janeiro, RJ, Brasil, 2025.

Clase latente	Perfil clínico predominante	Intervenciones prioritarias	S (%) IC95%	E (%) IC95%	F (%)
Clase 1	Bajo riesgo	Monitorear signos vitales; Orientar dieta equilibrada; Promover adhesión al tratamiento; Estimular vínculo familiar; Reforzar cuidados con la piel; Evaluar reposo y calidad del sueño;	82,5 (79,0–85,6)	87,2 (84,0–90,1)	36,8%
Clase 2	Riesgo medio	Incentivar adhesión al tratamiento como forma de reducción de sufrimiento futuro; Evaluar dolor y náuseas; Estimular autocuidado; Promover expresión emocional; Incentivar autoestima, autoconfianza y aceptación de la enfermedad; Ofrecer confort físico y psicológico;	85,3 (82,1–88,0)	89,0 (86,2–91,5)	41,5%
Clase 3	Alto riesgo	Gerenciar intercurrencias respiratorias y hemodinámicas; Regular diuresis, glicemia y PA; Intensificar vigilancia neurológica; Actuar en sufrimiento espiritual; Reforzar la importancia de la hemodiálisis regular para reducir riesgo de mortalidad.	90,1 (87,5–92,4)	92,0 (89,8–94,0)	21,7%

Abreviaturas: Se – sensibilidad; Es – especificidad; IC 95% – intervalo de confianza de 95%.

La Clase 1 concentró la mayor frecuencia de la muestra (36,8 %), lo que refleja el perfil predominante de pacientes en seguimiento regular y con buena adherencia terapéutica. La Clase 2 representó el 41,5 % de la población estudiada, lo que confirma la relevancia de las intervenciones educativas y de autocuidado para este grupo. Por su parte, la Clase 3, aunque menos prevalente (21,7 %), presentó los mayores índices de precisión, lo que indica la exactitud del modelo para identificar casos complejos y de mayor gravedad.

Este rendimiento global sugiere que el instrumento utilizado tiene una alta aplicabilidad clínica, lo que contribuye a una mayor resolución en la atención de personas en hemodiálisis. Debido al número limitado de páginas, la Tabla 3 presenta ejemplos de IE agrupados por clase latente, con sus respectivas medidas de rendimiento diagnóstico.

## DISCUSIÓN

Los resultados de este estudio demuestran que la aplicación del ACL con efectos aleatorios fue eficaz en la validación clínica de los indicadores del CIPE^®^, permitiendo la identificación de patrones diagnósticos con alta sensibilidad, especificidad y precisión global. Este enfoque estadístico refuerza la confiabilidad de los indicadores clínicos observables y su capacidad para discriminar perfiles latentes distintos, contribuyendo directamente a la calificación de la práctica diagnóstica en enfermería.

Estos resultados están en consonancia con un estudio que recopiló evidencias sobre la validación clínica de subconjuntos terminológicos del CIPE^®^ e identificó que, aunque la mayoría de las investigaciones aún se encuentran en una fase inicial, existe un reconocimiento cada vez mayor de la importancia de la validación clínica como etapa esencial para consolidar el lenguaje estandarizado en la práctica asistencial^(1)^.

Además, otro estudio destaca que la validación clínica contribuye a mejorar la calidad de la atención, al permitir que los términos utilizados reflejen con mayor precisión las necesidades reales de los clientes^(12)^. En el presente estudio, la asociación entre indicadores y clases latentes permitió no solo validar los términos, sino también agrupar intervenciones específicas por perfil clínico, lo que representa un avance metodológico en relación con investigaciones anteriores, que se centraron principalmente en la validación de contenido o semántica.

En el ámbito de las necesidades psicobiológicas, los enunciados de DE/RE e IE presentaron una alta consistencia clínica y solidez, como lo demuestran los altos índices de sensibilidad, especificidad e intervalos de confianza estrechos. Estos hallazgos concuerdan con un estudio metodológico que validó el subconjunto CIPE^®^ para personas en hemodiálisis, en el que la mayoría de los DE/RE e IE se centraron en las necesidades psicobiológicas, representando más del 70 % de los enunciados validados^(5)^.

Se observaron resultados similares en investigaciones dirigidas a la validación de intervenciones específicas, como el manejo del exceso de líquidos, que presentaron altos índices de validación, lo que refuerza la aplicabilidad clínica en la atención a pacientes en terapia renal sustitutiva. De este modo, el predominio de las necesidades psicobiológicas en los hallazgos de esta investigación confirma la centralidad de los aspectos fisiológicos en el proceso de atención a pacientes en hemodiálisis, determinantes para el mantenimiento de la seguridad clínica y la adherencia al tratamiento^(13)^.

También en este ámbito, se destacaron diagnósticos que reflejan complicaciones respiratorias, metabólicas y cardiovasculares, como “Disnea” y “Desequilibrio electrolítico”, cuya validación refuerza la importancia de la monitorización continua y de las intervenciones dirigidas a la estabilidad clínica. Otras afirmaciones, como “Ingesta nutricional deficiente”, “Estreñimiento” y “Sueño inadecuado”, ponen de manifiesto la importancia de los cuidados centrados en las dimensiones nutricional, gastrointestinal y del descanso, que a menudo se ven comprometidas debido a las restricciones alimentarias, la uremia y los síntomas asociados. También destacan “fatiga”, “riesgo de infección”, “integridad de la piel alterada”, “hipertensión arterial”, “anemia crónica” y “fiebre”, que representan afecciones de alta prevalencia en hemodiálisis y requieren estrategias de prevención, educación sanitaria y adherencia al tratamiento^(14)^.

El diagnóstico “Memoria deteriorada” puede reflejar alteraciones cognitivas derivadas de la cronicidad e influir en la adherencia a las orientaciones, lo que exige un mayor apoyo educativo. También se destacó el “Rendimiento sexual deteriorado”, a menudo descuidado en la práctica clínica, pero que interfiere en la autoestima, las relaciones interpersonales y la calidad de vida. Por último, el diagnóstico “No adherencia al tratamiento hemodialítico”, cuya comprensión va más allá de la mera asistencia a las sesiones, implica la aceptación, la motivación y el enfrentamiento de la condición crónica. El reconocimiento de estos aspectos amplía la perspectiva de la atención y refuerza la integralidad de la asistencia de enfermería a las personas en hemodiálisis^(15-17)^.

En el ámbito de las necesidades psicosociales, se verificó la validación de enunciados con consistencia clínica moderada, aunque menos prevalentes en relación con los psicobiológicos. Este hallazgo se aproxima a otros estudios que destacaron la presencia relevante, aunque minoritaria, de DE/RE vinculados a las necesidades psicosociales, representando aproximadamente el 20 % de los enunciados validados en subconjuntos terminológicos^(5)^.

Destacaron diagnósticos como “Proceso familiar perjudicado”, “Esparcimiento perjudicado” y “Comodidad perjudicada”, que reflejan las limitaciones impuestas por la rutina dialítica y los síntomas asociados, lo que compromete la convivencia familiar, el bienestar y la calidad de vida. A estos se suman “Autoestima negativa” y “Negación del tratamiento”, que revelan dificultades emocionales y de aceptación de la cronicidad, así como “Alfabetización en salud, perjudicada” y “Capacidad laboral, disminuida”, que evidencian las repercusiones sociales y educativas de la enfermedad, con impactos directos en la autonomía del autocuidado y en la productividad. Estos hallazgos refuerzan la necesidad de intervenciones educativas accesibles, apoyo psicológico y estrategias que favorezcan la reorganización de la vida cotidiana y la reinserción social.

Los pacientes en hemodiálisis suelen experimentar impactos emocionales, como ansiedad, fatiga y deterioro de la autoimagen, elementos que influyen directamente en su calidad de vida. El análisis de agrupamientos latentes aplicado a enfermedades crónicas mostró que los subgrupos de pacientes pueden diferenciarse por la gravedad de los síntomas psicosociales, lo que indica la importancia de intervenciones dirigidas también a estas necesidades^(18)^.

Así, la inclusión de enunciados psicosociales validados amplía la perspectiva del cuidado, yendo más allá de la dimensión fisiológica y fortaleciendo la integralidad de la asistencia^(19)^. Aunque en número reducido, la validación de enunciados relacionados con las necesidades psicoespirituales representa un avance en el reconocimiento de la dimensión espiritual en la atención a la persona en hemodiálisis^(20)^.

En el ámbito psicoespiritual, el diagnóstico “Sufrimiento espiritual” evidenció el peso de la dimensión existencial en el enfrentamiento de la enfermedad renal crónica. La dependencia de una terapia continua e invasiva puede intensificar los sentimientos de vacío, pérdida de sentido y vulnerabilidad, dificultando la adherencia y el enfrentamiento. La validación de esta afirmación apunta a la necesidad de prácticas de enfermería que incorporen la espiritualidad en la atención, valorando las creencias, los valores y los recursos internos del paciente como estrategias para fortalecer la resiliencia y promover una atención integral y humanizada.

En un estudio de validación clínica con pacientes en tratamiento conservador, diagnósticos como “Sufrimiento espiritual” y “Vínculo religioso perjudicado” se confirmaron como relevantes, lo que demuestra que la dimensión espiritual desempeña un papel importante en el enfrentamiento de la enfermedad renal crónica^(16)^. Aunque la proporción de estas afirmaciones sea pequeña, su incorporación al plan de asistencia revela la sensibilidad de la CIPE^®^ para abarcar la atención integral y humanizada, contemplando la experiencia existencial de los pacientes^(5,8,21)^.

Los hallazgos de este estudio presentan convergencias y contrastes relevantes cuando se comparan con la validación clínica de subconjuntos terminológicos de la CIPE^®^ dirigidos a personas con ERC en tratamiento conservador. La primera se refiere a la predominancia de DE/RE e IE relacionados con la adherencia al tratamiento, el control de la presión arterial y el manejo nutricional. Aunque estos elementos también están presentes en la atención de pacientes en hemodiálisis, el presente estudio evidenció especificidades adicionales, como el sufrimiento espiritual, la seguridad emocional y la sobrecarga clínica derivada de las complicaciones dialíticas. Además, la estratificación de los participantes por clases latentes permitió identificar patrones de atención distintos, lo que no se observó en los estudios con pacientes en tratamiento conservador. Estos contrastes refuerzan la importancia de adaptar los subconjuntos terminológicos a las realidades clínicas específicas, garantizando una mayor precisión en la identificación de las NHB y en la personalización de las intervenciones de enfermería^(9)^.

Por lo tanto, los resultados obtenidos en este estudio no solo validan clínicamente los indicadores del CIPE^®^, sino que también amplían su aplicabilidad al proponer una estrategia de estratificación diagnóstica y de intervención por perfil clínico. Esta propuesta representa una contribución innovadora a la práctica basada en la evidencia y al fortalecimiento del lenguaje profesional de la enfermería.

El presente análisis demostró la aplicabilidad de la Teoría de las NHB, junto con la ACL, como método robusto para la identificación de patrones diagnósticos en pacientes en tratamiento hemodialítico. La adopción de un modelo con tres clases latentes permitió la estratificación clínica de los pacientes en distintos niveles de complejidad, ofreciendo subsidios estadísticos relevantes para la planificación personalizada de la atención.

Las elevadas medidas de sensibilidad y especificidad observadas en diversos indicadores clínicos refuerzan su capacidad discriminatoria. Los indicadores asociados a la oxigenación, la regulación vascular, la nutrición y la eliminación presentaron una mayor consistencia en la Clase 3, lo que evidencia que los aspectos fisiológicos críticos tienden a concentrarse en pacientes con mayor compromiso asistencial. Por otro lado, los indicadores de los ámbitos psicosocial, emocional y espiritual mostraron un rendimiento adecuado, especialmente en la Clase 2, lo que demuestra que estas manifestaciones no son exclusivas de los casos graves y requieren una atención transversal en la práctica de la enfermería.

La agrupación de intervenciones por clase latente contribuye directamente a la práctica asistencial, al permitir la asociación entre el perfil clínico y el plan de cuidados individualizado. La composición de las intervenciones por clase sugiere que los pacientes de la Clase 1 se benefician de acciones educativas y preventivas; los de la Clase 2 requieren intervenciones de apoyo emocional, control sintomático y fortalecimiento del autocuidado; y los de la Clase 3 exigen planes complejos e interdisciplinarios para el manejo intensivo de las complicaciones clínicas y el sufrimiento psicosocial.

Así, los resultados refuerzan el potencial del enfoque multidimensional en la construcción de DE/RE e IE, evidencian la capacidad de la ACL para generar conocimiento clínico aplicable y señalan caminos prometedores para la consolidación de la práctica basada en la evidencia dentro de los sistemas clasificatorios de la CIPE^®^.

En cuanto a las limitaciones del estudio, la selección de una población específica puede haber reducido el alcance de los hallazgos, lo que dificulta su generalización. Además, el hecho de que el estudio se haya restringido a una sola región y contexto introdujo una característica cultural singular, que debe considerarse cuidadosamente en el análisis de los resultados, garantizando una mayor precisión en la interpretación de los hallazgos.

La implementación del subconjunto terminológico validado para personas en hemodiálisis requiere un enfoque estructurado e integrado en la rutina de los profesionales de enfermería. En primer lugar, es fundamental promover la formación continua para capacitar a los enfermeros en el uso correcto de los enunciados de DE/RE e IE de CIPE^®^. La realización de talleres y simulaciones clínicas puede contribuir a consolidar el razonamiento clínico necesario para identificar los indicadores específicos de los diagnósticos de este subconjunto y para tomar decisiones acertadas.

Otro aspecto esencial para la implementación exitosa del subconjunto terminológico es la creación de protocolos de atención basados en las definiciones validadas. La incorporación de estos términos en los manuales de conducta en hemodiálisis puede garantizar una mayor uniformidad en las prácticas de enfermería y reducir las variaciones en la conducción de los casos clínicos. Además, los criterios de validación pueden servir de base para auditorías internas, lo que permite supervisar el cumplimiento de las mejores prácticas e identificar oportunidades de mejora en la atención prestada.

Por último, la realización de estudios multicéntricos constituye un paso estratégico para ampliar la aplicabilidad del subconjunto validado en diferentes instituciones de salud. La replicación del modelo asistencial en otros servicios de hemodiálisis permitirá evaluar su eficacia en diversos contextos, reforzando su utilidad en la práctica clínica. Para garantizar la continuidad de la actualización, se recomienda que los profesionales involucrados participen en redes de colaboración científica, donde puedan compartir hallazgos y contribuir a la evolución del subconjunto terminológico del CIPE^®^.

La implementación eficaz de este subconjunto representa un avance significativo en la estandarización del lenguaje especializado de la enfermería en hemodiálisis, fortaleciendo la toma de decisiones de los profesionales, mejorando la seguridad del paciente y garantizando una atención más calificada a los usuarios.

## CONCLUSIÓN

La investigación evidenció la validación clínica de un subconjunto compuesto por 23 DE/RE y 169 IE de la CIPE^®^, dirigido al cuidado de personas con ERC en tratamiento hemodialítico. Los resultados de este estudio demuestran que la aplicación de la ACL constituye un enfoque metodológico sólido para la validación clínica de subconjuntos terminológicos de la CIPE^®^, especialmente en contextos marcados por la complejidad asistencial, como el cuidado de pacientes en hemodiálisis.

La identificación de tres clases latentes permitió diferenciar perfiles clínicos distintos, con altos niveles de sensibilidad, especificidad y precisión global, lo que refuerza la validez de los indicadores observables para fines diagnósticos. Con ello, este estudio contribuye a la consolidación del lenguaje estandarizado de la enfermería, fortalece la práctica basada en la evidencia y ofrece subsidios para la construcción de protocolos asistenciales más precisos, seguros e individualizados.

La aplicabilidad práctica del subconjunto se amplía al permitir la identificación temprana de riesgos, la planificación de intervenciones específicas y el monitoreo continuo de los resultados de enfermería. Se recomienda que futuras investigaciones exploren su implementación en diferentes contextos asistenciales, como la atención primaria, las unidades de terapia intensiva y los servicios de trasplante renal, además de investigar su impacto en la calidad de la atención, los resultados clínicos y la gestión de los servicios de salud.

## Data Availability

El conjunto de datos que respaldan las conclusiones de este estudio está disponible previa solicitud al autor correspondiente.
