# Supplementary material for: Erratum: Clinical validation of ICNP® terminological subset for people with chronic kidney disease undergoing hemodialysis
Source: Rev Esc Enferm USP. 2026 Mar 9;60:e20250364er. doi: 10.1590/1980-220X-REEUSP-2025-0364eren (PMC12970617; doi:10.1590/1980-220X-REEUSP-2025-0364eren)
Supplement: Translation

# Translation: Erratum: Validação clínica do subconjunto terminológico da CIPE^®^ para pessoas com doença renal crônica em hemodiálise

No artigo “Validação clínica do subconjunto terminológico da CIPE^®^ para pessoas com doença renal crônica em hemodiálise”, com número de DOI: https://doi.org/10.1590/1980-220X-REEUSP-2025-0364pt, publicado na Revista da Escola de Enfermagem da USP, 60:e20250364, 2026, na página 1:

**Onde se lia:**

“Juliana Otaciana da Silva”

**Leia-se:**

“Juliana Otaciana dos Santos”

Profa. Dra. Maria Amélia de Campos Oliveira

Editora-chefe

# Translation: Erratum: Validación clínica del subconjunto terminológico del CIPE^®^ para personas con enfermedad renal crónica en hemodiálisis

En el artículo “Validación clínica del subconjunto terminológico del CIPE^®^ para personas con enfermedad renal crónica en hemodiálisis”, con número de DOI: https://doi.org/10.1590/1980-220X-REEUSP-2025-0364es, publicado en la Revista da Escola de Enfermagem da USP, 60:e20250364, 2026, en la página 1:

**Donde se leía:**

“Juliana Otaciana da Silva”

**Lea:**

“Juliana Otaciana dos Santos”

Profa. Dra. Maria Amélia de Campos Oliveira

Editora-jefe
